# The TRIM21-FOXD1-BCL-2 axis underlies hyperglycaemic cell death and diabetic tissue damage

**DOI:** 10.1038/s41419-023-06355-1

**Published:** 2023-12-13

**Authors:** Wenwen Cheng, Cifeng Cai, Yifan Xu, Xueqi Xiao, Tiantian Shi, Yueling Liao, Xiaoyi Wang, Shasha Chen, Meiliang Zhou, Zhiyong Liao

**Affiliations:** 1 College of Life and Environmental Science, Wenzhou University, Wenzhou, 325035 China; 2 First Affiliated Hospital of Huzhou University, Huzhou, 313000 China; 3 Institute of Crop Sciences, Chinese Academy of Agricultural Sciences, Beijing, 100081 China

**Keywords:** Apoptosis, Diabetes, Ubiquitylation

## Abstract

Chronic hyperglycaemia is a devastating factor that causes diabetes-induced damage to the retina and kidney. However, the precise mechanism by which hyperglycaemia drives apoptotic cell death is incompletely known. Herein, we found that FOXD1, a FOX family transcription factor specifically expressed in the retina and kidney, regulated the transcription of BCL-2, a master regulator of cell survival. Intriguingly, the protein level of FOXD1, which responded negatively to hyperglycaemic conditions, was controlled by the TRIM21-mediated K48-linked polyubiquitination and subsequent proteasomal degradation. The TRIM21-FOXD1-BCL-2 signalling axis was notably active during diabetes-induced damage to murine retinal and renal tissues. Furthermore, we found that tartary buckwheat flavonoids effectively reversed the downregulation of FOXD1 protein expression and thus restored BCL-2 expression and facilitated the survival of retinal and renal tissues. In summary, we identified a transcription factor responsible for BCL-2 expression, a signalling axis (TRM21-FOXD1-BCL-2) underlying hyperglycaemia-triggered apoptosis, and a potential treatment for deleterious diabetic complications.

## Introduction

Diabetes mellitus (DM), which includes type 1 diabetes mellitus (T1DM) and type 2 diabetes mellitus (T2DM), is a metabolic and inflammatory disease and an increasing global health problem. DM is a leading cause of retinopathy, neuropathy, nephropathy, and macrovascular complications [1, 2]. Although T1DM and T2DM have distinct aetiologies [1, 3], their main clinical hallmark is hyperglycaemia, i.e., a chronic increase in the blood glucose level [1]. Numerous studies have indicated that hyperglycaemia plays a pivotal role in cell death and diabetic complications. Glycolysis, the main process for the metabolism of intracellular glucose, fails to fully dispose of intracellular glucose in patients with chronic hyperglycaemia [4], leading to the activation of several alternative glucose metabolic pathways, including the polyol, hexosamine, and protein kinase C (PKC) pathways [5]. The excess reactive oxygen species (ROS) produced via these pathways results in oxidative stress [4]. In diabetes, the presence of oxidative stress and accumulation of carbohydrates also facilitate the formation of advanced glycation end products (AGEs) [6], which initiate an inflammatory signalling cascade by binding to cell surface receptors such as the receptor for AGE (RAGE) [7]. ROS and oxidative stress disrupt the elaborate balances between antiapoptotic and proapoptotic BCL-2 family members on the mitochondrial outer membrane, resulting in the permeabilization of the membrane and the initiation of apoptosis [8, 9]. In addition, the activation of NF-κB signalling also plays a role in hyperglycaemia-induced apoptosis by stimulating the expression of cytokines such as TNFα [10]. However, the presence of other major signalling pathways that contribute substantially to hyperglycaemia-induced tissue injury has not been completely confirmed.

The BCL-2 family of proteins consists of three groups with distinct functions: antiapoptotic proteins, proapoptotic multidomain proteins, and proapoptotic BH3-only proteins [11]. Imbalances among these proteins result in the release of cytochrome *c* into the cytoplasm and the activation of the caspase cascade, which initiates apoptosis [12]. The abundance, stability, activity, and localization of BCL-2 family proteins contribute to cellular susceptibility to apoptosis [13] and are considerably involved in various physiological and pathological processes, such as cancers and drug resistance [11]. Regulation of mRNA transcription appears to be the dominant means of controlling the diverse expression levels of BCL-2 family proteins [13]. For example, p53 induces apoptosis by upregulating the transcription of mRNAs encoding proapoptotic proteins such as BAX, PUMA, and Noxa [14, 15] but repressing that of the mRNA encoding the antiapoptotic protein BCL-2 [13]. NF-κB and STAT3 drive the transcription of BCL-2 [16] and BFL1 [17] in the immune system to promote cell survival, thus maintaining immune homeostasis. Therefore, identifying a new transcription factor that responds to hyperglycaemia and activates the transcription of key players in the apoptotic process is clearly essential in understanding hyperglycaemia-induced tissue injury.

The FOX transcription factors, initially identified in *Drosophila melanogaster*, belong to the “winged helix” superfamily because they share a winged helix-turn-helix DNA-binding motif termed a “forkhead box” [18, 19]. They are evolutionarily conserved and regulate diverse biological processes ranging from developmental to physiological processes [18] and are involved in cancers [20–22], Parkinson’s disease [23], autism spectrum disorder [24], ocular abnormalities [25], immune dysregulation [26], and language acquisition deficiencies [27]. FOXD1, a transcription factor with known functions in renal and retinal development, is abundantly expressed in the retina, kidneys, central nervous system, and cardiomyocytes [19] and is coincidentally associated with severe organ damage resulting from chronic hyperglycaemia.

Recent reports have indicated that the abnormal overexpression of FOXD1 is related to tumorigenesis and the progression of several types of cancers [28–30]. The regulation of FOXD1 stability was uncharacterized until Zhang et al. reported that USP21 is a critical deubiquitinase of FOXD1 that removes its K48-linked polyubiquitin chains and stabilizes its protein to maintain the mesenchymal properties of glioblastoma stem cells [31]. However, the E3 ligases that can mediate the K48-linked polyubiquitination of FOXD1 are still unknown.

The flavonoids extracted from Tartary buckwheat, known as Tartary buckwheat flavonoids (TBFs), predominantly consist of rutin and quercetin and exhibit a wide range of bioactivities, such as antioxidant, antidiabetic, and anti-inflammatory activities [32]. TBFs have been shown to have significant antidiabetic effects, as evidenced by the marked reductions in serum glucose and insulin levels, improved insulin sensitivity, and alleviation of hyperglycaemia-induced oxidative stress [32]. The molecular mechanisms underlying these effects were reported to involve the inhibition of α-glucosidase and α-amylase activity [33, 34], thereby preventing the release of glucose into the bloodstream from the small intestine. Additionally, TBFs regulate the expression of PTP1B and PI3K to attenuate insulin resistance in muscle [35–37]. Furthermore, they enhance glucose uptake and decrease gluconeogenesis by regulating the expression of GLUT2, GLUT4, PEPCK and G6-Pase in the liver [35–37]. TBFs can also modulate the nuclear translocation of Nrf2 to upregulate the expression of antioxidative enzymes and ameliorate high glucose-induced oxidative stress [37]. However, whether TBF plays a non-antioxidant role in the regulation of high glucose-induced cell death is an important issue remaining to be addressed.

Herein, we unexpectedly found that FOXD1 plays a crucial role in regulating hyperglycaemia-induced apoptosis by directly modulating the transcription of BCL-2. Notably, FOXD1 proteins are downregulated under hyperglycaemic conditions, and their downregulation is controlled by TRIM21-mediated K48-linked polyubiquitination and subsequent proteasomal degradation. This unrecognized TRIM21-FOXD1-BCL-2 axis thus constitutes a novel molecular mechanism in hyperglycaemia pathogenesis. We also found that the FOXD1-BCL-2 axis is considerably involved in retinopathy and nephropathy in T2DM mice. Additionally, TBFs effectively reversed the downregulation of FOXD1 protein expression, thus restoring BCL-2 expression and protecting the kidney and retina from hyperglycaemic injury. In summary, we identified FOXD1 as a direct transcription factor for BCL-2 and identified a signalling axis (TRM21-FOXD1-BCL-2) underlying hyperglycaemia-induced cell apoptosis and tissue injury.

## Results

### Hyperglycaemia induces the apoptosis of venous, renal, and retinal cells

**Fig. 1:**
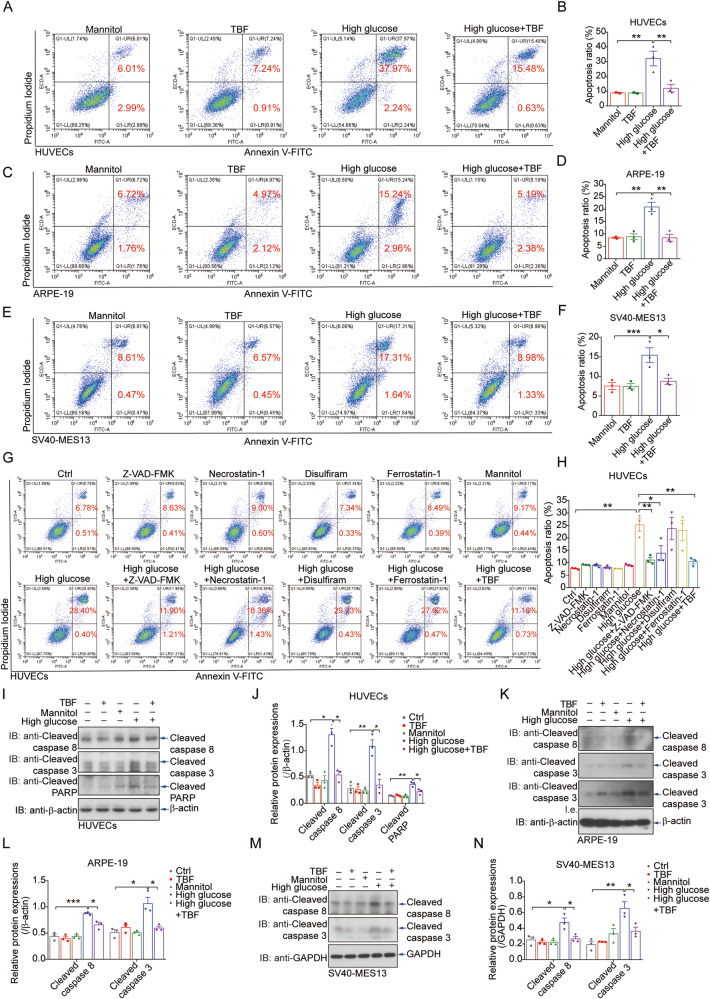
Hyperglycaemia induces the apoptosis of venous, renal, and retinal cells. Primary HUVECs cultured in high-glucose medium (33 mM) for 72 hours showed significant cell death, as revealed by propidium iodide (PI) staining via FACS analysis (**A**). Quantitative analysis of the apoptosis ratio is shown (**B**). Treatment with 5 μg/ml TBFs resulted in substantial protection against hyperglycaemia-induced HUVEC apoptosis. Mannitol was used as a negative control to eliminate the mild increase in osmotic pressure caused by high glucose. *n* = 3. **C**, **D** Hyperglycaemia-induced cell apoptosis was also apparent in ARPE-19 retinal epithelial cells, as revealed by PI staining via FACS analysis, and TBF protected against cell death. *n* = 3. **E**, **F** Hyperglycaemia-induced cell apoptosis was also apparent in SV40-MES13 mouse glomerular mesangial cells, as revealed by PI staining via FACS analysis, and TBF protected against cell death. *n* = 3. Inhibitors of apoptosis (Z-VAD-FMK), necroptosis (necrostatin-1), pyroptosis (disulfiram), and ferroptosis (ferrostatin-1) were individually incubated together with high glucose (33 mM) in HUVECs for 72 hours. Z-VAD-FMK and necrostatin-1 showed protective effects against hyperglycaemia-induced cell death (**G**); quantitative analyses of the cell death ratio (**H**). *n* = 3. High-glucose medium induced the production of cell apoptosis markers such as cleaved caspase 3, cleaved caspase 8, and cleaved PARP in primary HUVECs (**I**); quantitative analyses of the protein expression levels (**J**). *n* = 3. **K**, **L** High-glucose medium induced the production of cell apoptosis markers such as cleaved caspase 3 and cleaved caspase 8 in ARPE-19 cells. *n* = 3. **M**, **N** High glucose medium induced the production of cell apoptosis markers such as cleaved caspase 3 and cleaved caspase 8 in SV40-MES13 cells. *n* = 3. Scale bars = 200 μm. Unless otherwise specified, *n* = 3 independent experiments (mean ± SEM); *P < 0.05, **P < 0.01, ***P < 0.001, and ****P < 0.0001 by statistical analysis of the indicated comparison with ANOVA and the Bonferroni correction.

High-glucose medium induces apoptosis in venous endothelial, retinal and renal cells, constituting a primary mechanism for hyperglycaemic injury in diabetes patients [2, 4, 38, 39]. High glucose-induced cell death was confirmed in primary human umbilical vein endothelial cells (HUVECs) by both an annexin V-FITC/PI apoptosis assay (Fig. 1A, B) and propidium iodide (PI) staining (Supplementary Fig. 1A, B). Additionally, similar effects were observed in ARPE-19 retinal epithelial cells (Fig. 1C, D, Supplementary Fig. 1C, D) and SV40-MES13 glomerular mesangial cells (Fig. 1E, F, Supplementary Fig. 1E, F). Intriguingly, TBFs, the antioxidant compounds extracted from the provincial food buckwheat (Supplementary Fig. 1G–J), robustly protected these cells from death, as evidenced by the reduced numbers of dead cells observed by FACS and PI staining (Fig. 1A, F, Supplementary Fig. 1A–F). To differentiate the diverse death programs involved in high glucose-induced cell death, we examined these cells for markers of apoptosis, pyroptosis, ferroptosis, and necroptosis. FACS analysis combined with pathway inhibitor treatment revealed the induction of both programmed apoptotic death and necroptosis under high-glucose stress; apoptosis accounted for most of the cell death triggered by hyperglycaemia in HUVECs (Fig. 1G, H), ARPE-19 cells (Supplementary Fig. 1K-L), and SV40-MES13 cells (Supplementary Fig. 1M, N). The induction of apoptosis in primary HUVECs was further validated by immunoblot analysis of caspases and PARP (Fig. 1I, J). Similarly, changes in caspase levels were also observed in ARPE-19 (Fig. 1K, L) and SV40-MES13 (Fig. 1M, N) cells. These observations suggest that a high-glucose environment triggers the death of venous, retinal, and renal epithelial cells via the apoptotic pathway and that a compound extracted from a food product exerts a potential protective effect against this process.

### Hyperglycaemia decreases the protein level of FOXD1

**Fig. 2:**
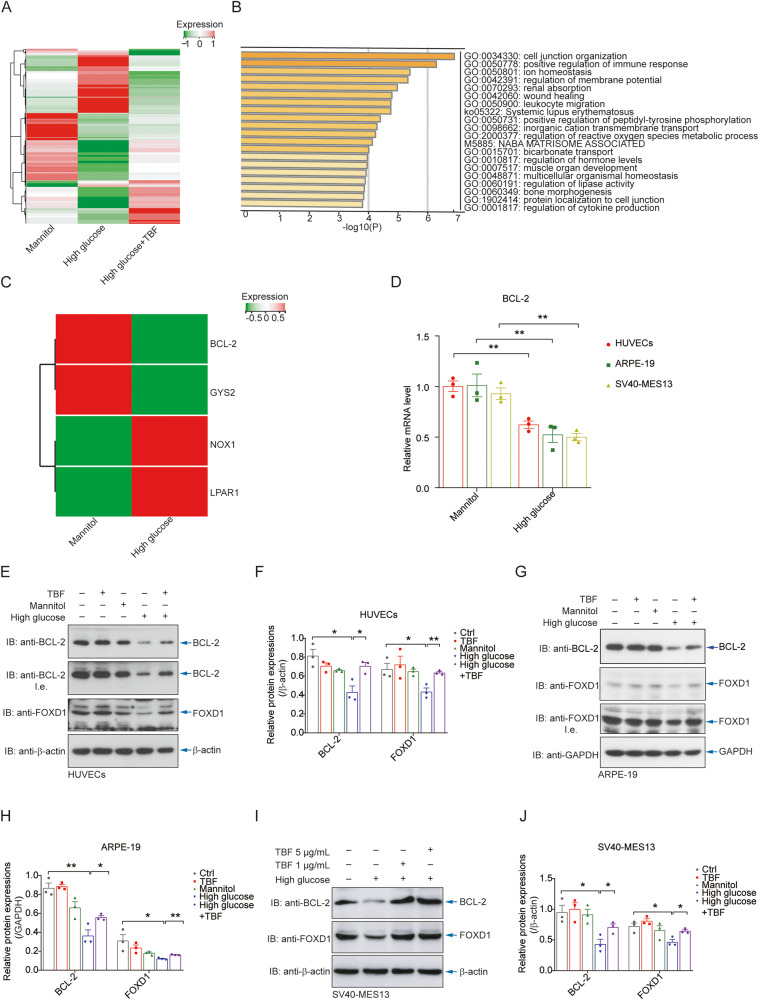
Hyperglycaemia decreases the protein level of FOXD1. **A** Transcriptome sequencing of HUVECs cultured in high-glucose medium and treated with TBFs was performed, indicating the alteration of multiple genes. **B** Gene annotations illustrated the alteration of multiple pathways under hyperglycaemia, particularly for those relating to cell junction organization and immune response. **C** The results of the transcriptome assay illustrated that the high-glucose environment markedly reduced BCL-2 mRNA expression. **D** RT‒qPCR analysis validated the results of the transcriptome analysis for BCL-2 expression in venous, retinal, and renal cells. *n* = 3. Immunoblotting results indicated that BCL-2 and FOXD1 proteins in HUVECs were downregulated under high-glucose conditions and that treatment with TBFs partially restored this regulation (**E**). Quantitative analyses of the relative protein expression levels are shown (**F**). *n* = 3. **G**, **H** Immunoblotting results indicated that BCL-2 and FOXD1 proteins in ARPE-19 cells were downregulated under high-glucose conditions. *n* = 3. **I**, **J** Immunoblotting results indicated that BCL-2 and FOXD1 proteins in SV40-MES13 cells were downregulated under high-glucose conditions. *n* = 3.

To understand the mechanism underlying high glucose-induced cell death and the preventive effect of TBFs, we performed transcriptome sequencing of primary human HUVECs after the indicated treatment, and the results indicated alterations in the expression of multiple genes (Fig. 2A), particularly those related to cell junction organization and immune response pathways (Fig. 2B). In addition to genes previously reported [40–42], BCL-2 mRNA expression was markedly reduced by the high-glucose environment (Fig. 2C). The RT‒qPCR results validated the results of the transcriptome analysis for BCL-2 expression (Fig. 2D). Notably, the results of immunoblotting confirmed the downregulation of BCL-2 and FOXD1 protein expression in HUVECs (Fig. 2E, F), retinal epithelial cells (Fig. 2G, H), and glomerular mesangial cells (Fig. 2I, J) under high-glucose conditions, and this downregulation was significantly reversed upon TBF treatment. Interestingly, the mRNA expression levels of BCL-XL and MCL-1, two antiapoptotic members of the BCL-2 family, were decreased in response to high-glucose treatment; however, their expression levels were restored by TBF treatment (Supplementary Fig. 2A). Additionally, the mRNA expression of BAX, a proapoptotic member of the BCL-2 family, was upregulated in response to high-glucose conditions and subsequently restored upon treatment with TBFs (Supplementary Fig. 2B). These observations suggest the plausible regulation of FOXD1 and BCL-2 expression in response to a high-glucose environment.

### FOXD1 binds to the BCL-2 promoter and promotes BCL-2 transcription

**Fig. 3:**
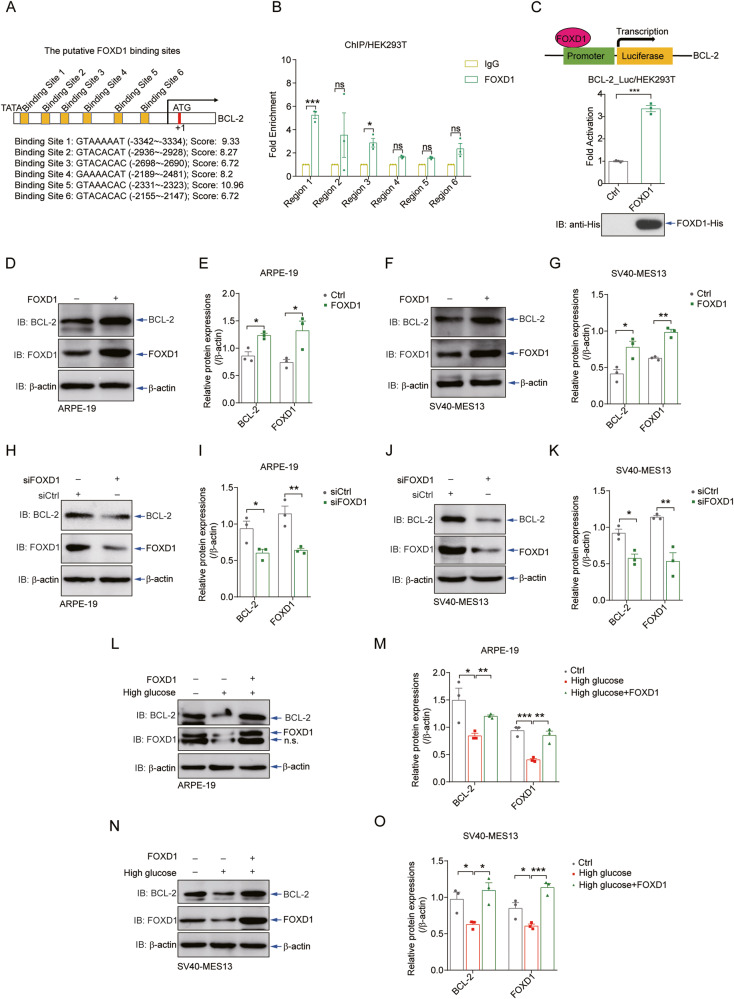
FOXD1 binds to the BCL-2 promoter and promotes the transcription of BCL-2. **A** Six putative FOXD1-binding sites were predicted by https://jaspar.genereg.net on the promoter of human BCL-2. **B** ChIP assays performed with HEK293T cells after anti-FOXD1 immunoprecipitation validated the binding of FOXD1 to the human BCL-2 promoter (bp -3462 to -1462). Six putative binding sites are located in these six regions, each of which contains approximately 200 bp. **C** A reporter assay showed that FOXD1 significantly potentiated the activation of the BCL-2 promoter, which contains the known BCL-2 promoter sequence located 2147–3547 bp upstream of the translation start site. *n* = 3. The delivery of FOXD1 into ARPE-19 (**D**, **E**) and SV40-MES13 (**F**, **G**) cells by the lentiviral system resulted in a significant increase in BCL-2 protein levels. Quantitative analyses of relative protein expression are shown (**E**, **G**). *n* = 3. siRNA-mediated depletion of FOXD1 in APRE-19 (**H**, **I**) and SV40-MES13 cells (**J**, **K**) affected the protein levels of BCL-2. *n* = 3. Ectopic expression of FOXD1 in these cells by lentivirus rescued the high glucose-induced downregulation of BCL-2 expression in APRE-19 (**L**, **M**) and SV40-MES13 (**N**, **O**) cells. *n* = 3.

We thus searched for the binding motif of FOXD1 (GTAAACAC) within the promoters of the apoptosis regulators at https://jaspar.genereg.net. Intriguingly, BCL-2, the master regulator of cell survival, had six consecutively potential binding elements for FOXD1 in its promoter (Fig. 3A). A ChIP assay using the FOXD1 immunoprecipitate confirmed the binding of FOXD1 to the BCL-2 promoter in regions 1 and 3, which contained binding sites 1 (GTAAAAAT) and 3 (GTACACAC) (Fig. 3B). In a reporter assay, overexpression of FOXD1 significantly increased the activity of the BCL-2 promoter (Fig. 3C), which contained the known BCL-2 promoter sequence located 2147-3547 bp upstream of the translation start site. These observations suggest the positive regulation of BCL-2 mRNA expression by the transcription factor FOXD1.

To examine whether FOXD1 regulates the expression of endogenous BCL-2 in retinal and renal cells, we generated a lentiviral system to express FOXD1 in the ARPE-19 and SV40-MES13 cell lines. Delivery of FOXD1 into these cells was confirmed and resulted in a significant increase in the BCL-2 protein level (Fig. 3D–G). In contrast, siRNA-mediated depletion of endogenous FOXD1 decreased the protein level of BCL-2 in these cells (Fig. 3H–K). Notably, lentivirus-mediated ectopic expression of FOXD1 in these cells rescued the high glucose-induced downregulation of BCL-2 expression (Fig. 3L–O). Therefore, these data suggest that FOXD1 binds to the BCL-2 promoter and promotes BCL-2 transcription during hyperglycaemia.

### TRIM21 ubiquitinates FOXD1 and drives its degradation in response to hyperglycaemia

**Fig. 4:**
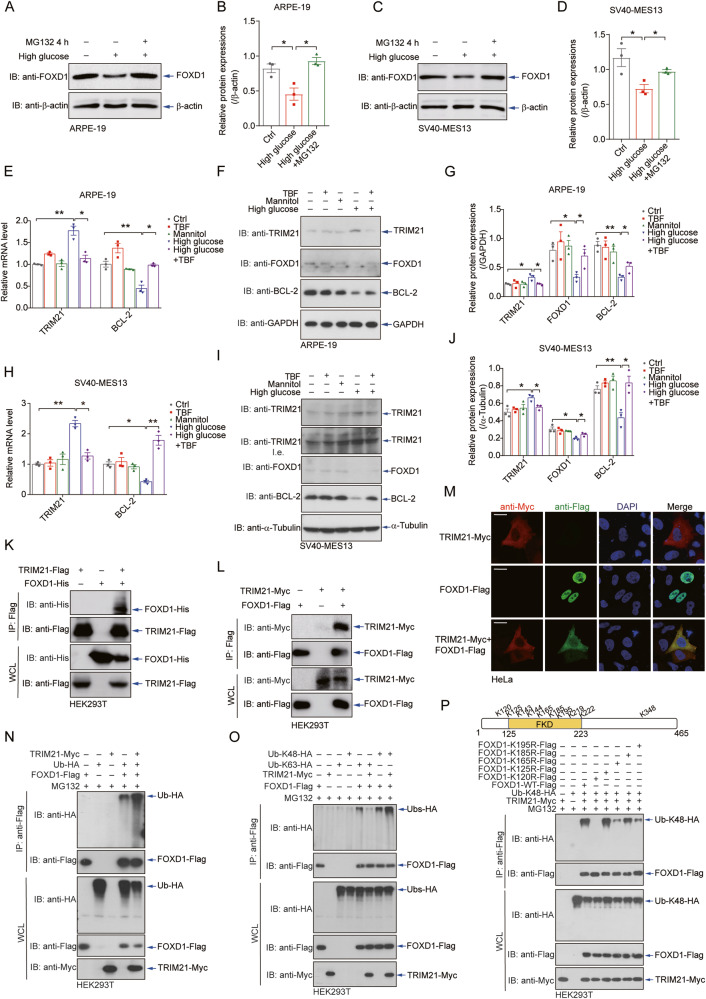
TRIM21 ubiquitinates FOXD1 and drives its degradation in response to hyperglycaemia. Treatment with 10 μM MG132 for 4 hours attenuated the high glucose-induced downregulation of FOXD1 protein expression in ARPE-19 (**A**, **B**) and SV40-MES13 (**C**, **D**) cells. *n* = 3. Culture in high-glucose medium increased both the mRNA (**E**) and protein (**F**) levels of TRIM21 in ARPE-19 cells, and this effect was reversed by TBF treatment. Quantitative analyses of relative protein expression are shown (**G**). *n* = 3. Culture in high-glucose medium increased both the mRNA (**H**) and protein (**I**) levels of TRIM21 in SV40-MES13 cells, and this effect was reversed by TBF treatment. Quantitative analyses of relative protein expression are shown (**J**). *n* = 3. **K**, **L** Coimmunoprecipitation assays using distinct tags revealed an interaction between FOXD1 and TRIM21. *n* = 3. **M** Immunofluorescence staining revealed colocalization of TRIM21 and FOXD1 in the cytoplasm. FOXD1 was evidently exported from the nucleus in the presence of TRIM21. Scale bars = 20 μm. *n* = 3. **N** Polyubiquitination of FOXD1 was detected when TRIM21 was cotransfected with FOXD1 in HEK293T cells in the presence of ubiquitin proteins. *n* = 3. **O** TRIM21 promoted K48-linked ubiquitination but suppressed K63-linked ubiquitination of FOXD1 in HEK293T cells. *n* = 3. **P** TRIM21-Myc was cotransfected with wild-type or the indicated mutant FOXD1s into HEK293T cells for 24 hours, revealing that K120, K165, and K195 were the primary residues required for TRIM21-mediated modification or interaction. *n* = 3.

In retinal and renal epithelial cells, the high glucose-induced downregulation of FOXD1 protein expression was blocked by treatment with MG132 (Fig. 4A–D), a specific proteasome inhibitor. To investigate the high glucose-induced instability of FOXD1, we expressed Flag-tagged FOXD1 in HEK293T cells in the presence of ubiquitin and conducted immunoprecipitation followed by mass spectrometry. TRIM21, a member of the tripartite motif (TRIM) family of E3 ligases, was among the proteins that interacted with FOXD1 (Supplementary Fig. 3A). Intriguingly, the mRNA and protein expression levels of TRIM21 in ARPE-19 cells (Fig. 4E–G), SV40-MES13 cells (Fig. 4H–J), and HUVECs (Supplementary Fig. 3B–D) were upregulated during high-glucose treatment but returned to basal levels after treatment with TBFs, suggesting that TRIM21 might be involved in the high glucose-induced regulation of FOXD1 instability. Furthermore, notably, the upregulation of TRIM21 expression and the degradation of FOXD1 appeared to be specifically triggered by high glucose levels rather than oxidative stress induced by H_2_O_2_ (Supplementary Fig. 3E–J). Similarly, the increase in the expression of TRIM21 triggered by high-glucose exposure was not affected by diabetes-related AGEs and ROS, as demonstrated through the use of the inhibitors FPS-ZM1 (RAGE) and setanaxib (ROS) (Supplementary Fig. 3K, L).

The co-immunoprecipitation of differentially tagged FOXD1 and TRIM21 in HEK293T cells confirmed their interaction (Fig. 4K, L). Surprisingly, immunofluorescence imaging revealed that FOXD1, a transcription factor localized exclusively in the nucleus, was exported from the nucleus, and its signal overlapped with that of TRIM21 in the cytoplasm (Fig. 4M), providing strong evidence for the interaction and potential modifications of FOXD1 and TRIM21.

We then performed a ubiquitination assay with HEK293T cells in the presence of ubiquitin proteins, detecting a robust signal indicating the TRIM21-mediated promotion of FOXD1 polyubiquitination (Fig. 4N). Further analyses utilizing K48-linked or K63-linked ubiquitin indicated the presence of both K48- and K63-linked ubiquitin chains on FOXD1, but TRIM21 increased only the K48-linked ubiquitination of FOXD1 (Fig. 4O). We then attempted to identify the lysine residues responsible for this K48-linked ubiquitination by mutating all lysine residues into arginine (K-to-R) individually and examining their ubiquitination status (Fig. 4P). The K120R, K165R, and K195R mutants displayed significantly compromised ubiquitination, suggesting that these residues are required for TRIM21-mediated modifications or interactions. Collectively, these observations suggest the occurrence of high glucose-induced expression of TRIM21, the TRIM21-mediated K48-linked ubiquitination of FOXD1, and the TRIM21-mediated control of FOXD1 stability.

### TBFs protect against hyperglycaemic injury in mice by regulating the FOXD1-BCL-2 axis

**Fig. 5:**
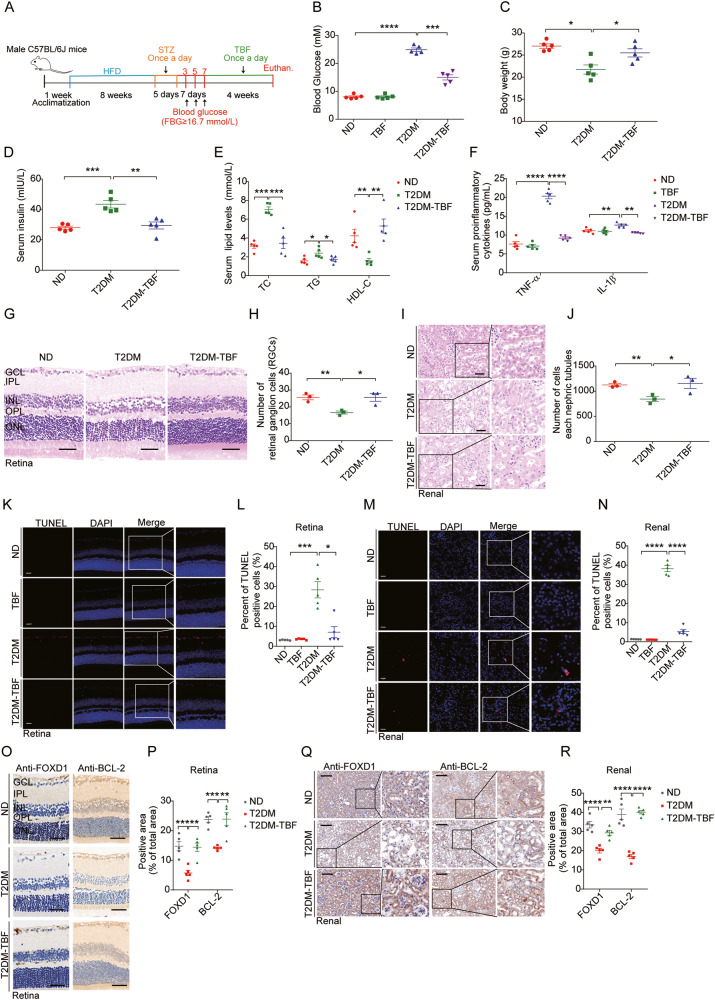
TBFs protect against hyperglycaemic injury in mice by regulating the FOXD1-BCL-2 axis. **A** Schematic figures showing the design, establishment, and validation of the murine T2DM model. Blood glucose (**B**), body weight (**C**), serum insulin (**D**), and serum total cholesterol/triglyceride/HDL-C (**E**) were measured in T2DM mice treated with or without TBFs. *n* = 5. **F** Serum TNF-α and IL-1β were measured by ELISA in T2DM mice treated with or without TBFs. *n* = 5. Representative images of H&E staining of the retina (**G**) and renal tubule (**I**). Quantitative analyses of retinal ganglion cells (**H**) and cell numbers in the renal tubule (**J**) are shown. Scale bars=50 μm. *n* = 5. The TUNEL assay indicated that cell death was increased in retinas (**K**, **L**) and kidneys (**M**, **N**) with hyperglycaemic injury. TBF treatment reversed this effect. Quantitative analyses of cell death in the retina (**L**) and kidney (**N**) are shown. Scale bars = 100 μm. *n* = 5. IHC staining indicated that the FOXD1 (**O**, **P**) and BCL-2 (**Q**, **R**) protein levels were decreased in retinas and kidneys with hyperglycaemic injury. TBF treatment reversed this effect. Scale bars = 100 μm (left panels of **G**, **O**, and **Q**) or scale bars = 50 μm (left panels of **I**, **K**, and **M**). *n* = 5.

To evaluate the physiological role of the FOXD1-BCL-2 axis in regulating hyperglycaemic organ damage, we generated a murine T2DM model by continuous high-fat diet (HFD) feeding and treatment with streptozotocin (Fig. 5A). This murine model was successfully established, and the model mice exhibited significantly elevated blood glucose levels (Fig. 5B, Supplementary Fig. 4A) and decreased body weights (Fig. 5C, Supplementary Fig. 4B), in addition to increased serum levels of insulin (Fig. 5D), total cholesterol (TC), triglycerides (TG), and decreased high-density lipoprotein cholesterol (HDL-C) (Fig. 5E). Furthermore, T2DM mice displayed significantly elevated proinflammatory cytokines (Fig. 5F, Supplementary Fig. 4C). Treatment with TBFs significantly protected against hyperglycaemic injuries, with substantially downregulated T2DM phenotypes in murine blood (Fig. 5B–F, Supplementary Fig. 4C). Examination of retinal and renal tissue samples from T2DM mice revealed hyperglycaemic damage to both organs, including the loss of ganglion cells in the retina (Fig. 5G, H) and a decreased cell number in each renal tubule (Fig. 5I, J). TBF treatment led to the recovery of the phenotypes (Fig. 5G–J). The incidence of cell death in the retina and renal tissue samples from T2DM mice, as detected by a terminal deoxynucleotidyl transferase dUTP nick end labelling (TUNEL) assay (Fig. 5K–N) and immunohistochemical (IHC) staining for activated caspase 3 (cleaved caspase 3) (Supplementary Fig. 4D–G), was significantly increased. However, this phenotype was effectively ameliorated by TBF treatment (Fig. 5K–N, Supplementary Fig. 4D–G). Furthermore, reduced levels of FOXD1 and BCL-2 proteins were observed in both retinal and renal tissue samples through IHC staining (Fig. 5O–R) and immunoblotting (Supplementary Fig. 4H–K). Notably, treatment with TBFs restored these protein levels (Fig. 5O–R, Supplementary Fig. 4H–K). Collectively, the results of the animal experiments validated the presence of hyperglycaemia-induced FOXD1-BCL-2 regulation and the beneficial effects of TBFs on hyperglycaemic injury through the regulation of this signalling axis.

## Discussion

**Fig. 6:**
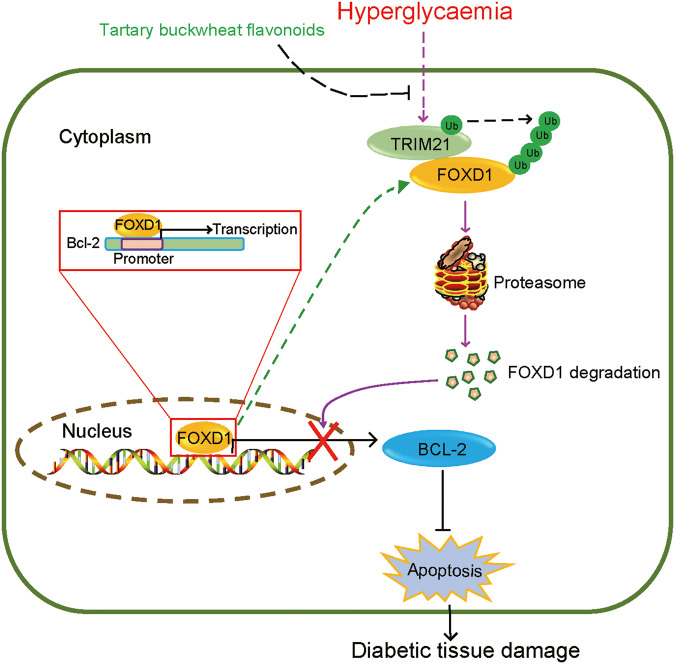
Hyperglycaemia induces cell apoptosis via the TRIM21-FOXD1-BCL-2 signalling axis. Chronic hyperglycaemia induces the expression of the ubiquitin E3 ligase TRIM21, which polyubiquitinates the transcription factor FOXD1 to control its protein stability, leading to hyperglycaemia-triggered downregulation of FOXD1 proteins. FOXD1 promotes the transcription of the antiapoptotic gene BCL-2 by directly binding to the BCL-2 promoter. Accordingly, hyperglycaemia-triggered degradation of FOXD1 mediated by TRIM21 shifts the balance between proapoptotic and antiapoptotic proteins and initiates apoptosis and tissue injury.

DM is a global metabolic and inflammatory disease caused by chronic hyperglycaemia and is associated with cell death, tissue injury, and diabetic complications. The overproduction of ROS and AGEs, induced by chronic hyperglycaemia, disrupts the elaborate balance of BCL-2 family proteins and permeabilizes the mitochondrial outer membrane to initiate intrinsic apoptosis. However, it is still not completely known whether the presence of other pathways substantially contributes to hyperglycaemia-induced cell death and tissue injury. Herein, we unexpectedly found that the E3 ligase TRIM21 interacted with and polyubiquitinated the transcription factor FOXD1 to control its protein stability under hyperglycaemic conditions, leading to hyperglycaemia-induced downregulation of FOXD1. We revealed that FOXD1 promoted the mRNA transcription of the antiapoptotic mediator BCL-2 by binding to the BCL-2 promoter at a conserved motif (GTAAAAAT). As a result, hyperglycaemia-induced degradation of FOXD1 shifts the balance to the proapoptotic state and initiates mitochondrial outer membrane permeabilization (MOMP) (Fig. 6). This previously unrecognized TRIM21-FOXD1-BCL-2 axis is a novel and intriguing mechanism underlying the pathogenesis of hyperglycaemia, and its participation has been validated in retinopathy and nephropathy in T2DM mice. Additionally, we found that TBFs, compounds extracted from the provincial food buckwheat, interfere with this cascade and effectively protect against hyperglycaemia-induced injury by preventing FOXD1 degradation. Accordingly, TBFs protect against renal and retinal damage from hyperglycaemic injury. Collectively, our findings provide evidence that FOXD1 is a new transcriptional regulator of BCL-2 under hyperglycaemic conditions and that an unrecognized TRIM21-FOXD1-BCL-2 axis transduces signalling related to hyperglycaemia-induced cell apoptosis and tissue injury.

Hyperglycaemia-induced cell death, including apoptosis and necroptosis, has been observed in various cell types associated with diabetic complications [43]. Recent research findings indicate that a high glucose level induces a transition from extrinsic apoptosis to necroptosis, thereby contributing to the development of diabetic kidney disease, which is dependent on mitochondrial ROS and glycolysis [44–46]. Interestingly, we also detected necroptosis during high-glucose treatment and found that TBF significantly prevented high glucose-induced cell death (Fig. 1G, H, Supplementary Fig. 1K–N). Given the potent antioxidant effects of TBFs [32], it is worthwhile to investigate their potential role in high glucose-induced necroptosis.

The intrinsic apoptosis pathway, regulated by members of the BCL-2 family, has been demonstrated to be involved in high glucose-induced cellular apoptosis [47]. BCL-2 is a prototypical member of the BCL-2 family of proteins, and its regulation is mediated primarily by its mRNA transcription and cellular localization [48]. Interactions between proapoptotic and antiapoptotic BCL-2 family members on the surface of mitochondria control the intrinsic apoptosis pathway and maintain a delicate balance between cell survival and death [48], which is crucial for proper tissue development and homeostasis and plays a role in numerous physiological processes from development to various human diseases [13]. To date, several transcription factors have been shown to control the activity of the BCL-2 promoter, for example, Aiolos in IL-2-deprived T cells [49], Mitf in the context of melanoma cell viability [50], and Pi in the context of B-cell differentiation [51]. Furthermore, mutations [52], posttranscriptional mechanisms [53], and posttranslational mechanisms [52, 54] are also involved in the dysregulation of BCL-2 family members in various haematopoietic cells and cancers. The identification of FOXD1, a retina- and renal-enriched transcription factor, substantially advances of our understanding of regulating apoptotic pathways in response to high blood glucose levels.

In addition, we observed that TBFs can reversed the decreases in the mRNA levels of BCL-XL and MCL-1, two antiapoptotic members of the BCL-2 family, and the increase in the mRNA level of BAX, a proapoptotic member of the BCL-2 family [55, 56], induced by high-glucose treatment (Supplementary Fig. 2A, B). However, the underlying mechanism of this phenotype remains unelucidated. It remains to be determined whether TBF regulates BCL-XL, MCL-1, or BAX transcription in a similar manner as it regulates BCL-2 transcription or whether it exerts its influence through the modulation of other signalling pathways associated with high glucose-induced apoptosis, such as pathways mediating mitochondrial function [57] or MAPK cascades [47].

FOXD1, which belongs to the forkhead-winged helix family, is a crucial transcription regulator in renal [58, 59] and ocular [60] development, mitochondrial activity [61], cell differentiation [61], and cell reprogramming [62]. Coincidently, the retina and kidney are tissues/organs susceptible to hyperglycaemic injury, as frequently observed in T2DM patients [2, 38, 39]. In addition to FOXD1, other Fox family members have been reported to regulate apoptotic processes under diverse physiological and pathological conditions, as exemplified by the upregulation of Bim by FOXOs [63, 64]. Overexpression of FOXD1 is related to abnormal cell proliferation and occurs in numerous human cancers, including cancers of the prostate, lung, and liver [19]. Physiological and pathological studies on the role of the FOXD1-BCL-2 axis may provide insights into tumour occurrence and progression. Our investigations suggest the organ- and context-dependent regulation of BCL-2 and its physiological implications during T2DM development. Furthermore, the finding that FOXD1 plays a vital role in hyperglycaemia-induced renal and retinal injury implies the therapeutic potential of targeting the FOXD1-BCL-2 axis to attenuate the progression of T2DM. The progressive failure and reduction in the number of insulin-secreting pancreatic β-cells are significant factors contributing to T2DM progression [65]. Recent research findings have elucidated the physiological roles of various BCL-2 family proteins in regulating β-cell function and apoptosis through the modulation of mitochondrial metabolism, glucose responsiveness, ROS signalling [66, 67], and even ER stress-related unfolded protein response (UPR) signalling [68]. Although FOXD1 is overexpressed in pancreatic cancer cells and facilitates the progression of pancreatic cancers [69], the expression and physiological role of FOXD1 in β-cells remain unknown. Investigating the role of the FOXD1-BCL-2 axis in β-cell apoptosis associated with hyperglycaemia holds significant scientific interest.

The regulation of FOXD1 protein was recently described. Zhang et al. reported that USP21, a deubiquitinase of FOXD1, removes K48-linked polyubiquitin chains from FOXD1 and maintains its stability [31]. However, the E3 ligases of FOXD1 are still unknown. Through this study, we revealed that FOXD1 is polyubiquitinated by the RING E3 ligase TRIM21, which leads to its proteasomal degradation. TRIM21 belongs to the TRIM family and contains a RING domain with E3 ubiquitin ligase activity [70], functioning as an essential regulator of immunity [71–74] and cancer [75, 76]. Intriguingly, TRIM21 negatively regulates glucose metabolism in human cancers by modulating the degradation of regulators to promote the dysregulation of glycolysis, for example, promoting the degradation of PFKP [77] and G6PD [78]. However, very little is known about how the protein level of TRIM21 is regulated. Notably, we found that the mRNA and protein levels of TRIM21 were both increased under hyperglycaemic conditions. Interestingly, these changes in TRIM21 expression were independent of hyperglycaemia-induced oxidative stress. However, the mechanism of this regulation is still unknown but is possibly related to the PI3K/AKT pathway, which is considerably associated with glucose metabolism and insulin resistance [79]. Furthermore, the upregulation of TRIM21 was reversed by TBF treatment, suggesting a potential non-antioxidant role for TBFs in regulating high glucose-induced cell death.

In conclusion, we revealed a new regulatory loop of hyperglycaemia-induced cell injury, validated this TRIM21-FOXD1-BCL-2 axis in a murine T2DM model, and suggested the protective role of TBFs in preventing diabetes-induced organ injury by interfering with this cascade. The identification of FOXD1 as a critical transcription factor for BCL-2 in the retina and kidney and the TRIM21-mediated regulation of FOXD1 stability complement our findings regarding therapeutic strategies for diabetes mellitus.

## Materials and methods

### Expression plasmids, viruses, reagents, antibodies and mice

Expression plasmids encoding Myc-, Flag-, His- or HA-tagged wild-type and mutant human FOXD1, TRIM21, Ub, K48-Ub, and K63-Ub, in addition to reporter vectors containing the BCL-2 promoter, were purchased from RiboBio. Lentivirus-FOXD1 was obtained from Taitool. All coding sequences were verified by DNA sequencing, and detailed information on the plasmids is provided in Supplementary Table 1.

The pharmacological reagents MG132 (Selleck) and puromycin (Yeasen) were purchased and used in accordance with the manufacturers’ instructions. Referencing a previous report [80], Tartary buckwheat flavonoid (TBF) extracts were purified by AB-8 macroporous adsorption resin (M0042, Solarbio) and identified by HPLC‒MS. Z-VAD-FMK (S7023), disulfiram (S1680), ferrostatin-1 (S7243), necrostatin-1 (S8037), FPS-ZM1 (S8185), and setanaxib (S7171) were purchased from Selleck. Carboxymethylcellulose sodium salt (9004-32-4) and streptozocin (STZ, 18883-66-4) were purchased from Sigma‒Aldrich (USA). High-fat rodent chow (60% from fat) was purchased from Research Diets, Inc. (New Brunswick, Canada).

The anti-HA (3724 S), anti-Flag (14793 S), anti-Myc (2276 S), anti-His (12698 S), anti-cleaved PARP (5625 S), anti-cleaved caspase 3 (9664 S), anti-cleaved caspase 8 (9496 S), anti-GAPDH (5174 S), and anti-TRIM21 (92043 S) monoclonal antibodies were purchased from Cell Signaling Technology. The anti-Caspase 8 antibody (66093-1-Ig) was purchased from Proteintech. The anti-FOXD1 (ab49156), anti-BCL-2 (ab182858), and anti-TRIM21 (ab207728) antibodies were obtained from Abcam. The anti-FOXD1 antibody (D260349) was obtained from Sangon Biotech. The anti-FOXD1 antibody (PA5-35145) was purchased from Thermo. The anti-β-actin antibody (AA128) was purchased from Beyotime Biotechnology. Normal anti-mouse IgG (7076 S) and anti-rabbit IgG (7074 S) were purchased from Cell Signaling Technology. Detailed information on all the antibodies used for immunoblotting, immunoprecipitation, and immunofluorescence staining is provided in Supplementary Table 2.

Wild-type C57BL/6 J mice were ordered from Shanghai SLAC Laboratory Animal Co., Ltd. The type II diabetes mellitus mouse model was generated following a previously published method [81]. A control diet and a HFD purchased from Research Diets Incorporated Company were used to feed wild-type C57BL/6 J mice.

### Cell culture, transfections, and infections

ARPE-19, SV40-MES13, HEK293T, and HeLa cells were obtained from ATCC. Primary HUVECs were a gift from Dr. Xiaoqing Yan (Wenzhou Medical University). No cell lines used in this study were found in the database of commonly misidentified cell lines maintained in the ICLAC and National Center for Biotechnology Information (NCBI) BioSample database. Cell lines were frequently checked for morphology under a microscope and tested for mycoplasma contamination but were not authenticated. HEK293T and HeLa cells were maintained in DMEM containing 10% foetal bovine serum (FBS) at 37 °C in 5% CO_2_ (v/v). ARPE-19 and SV40-MES13 cells were cultured in DMEM/F12 containing 10% foetal bovine serum (FBS) at 37 °C in 5% CO2 (v/v). ARPE-19 and SV40-MES13 cells with stable FOXD1 expression were generated by transducing a lentiviral vector followed by the ORF of FOXD1 and selection with puromycin (1.5 μg/ml) for 72 hours. Polyethylenimine (PEI, Polysciences) transfection reagent was used for plasmid transfection. siRNA transfection was performed using Lipofectamine RNAiMAX (Invitrogen) reagent/Lipofectamine 2000 (Invitrogen) reagent.

### Cell death analysis by PI staining

PI (Sigma‒Aldrich, P4170) staining was used to quantify cell death. In brief, HUVECs treated with mannitol (27.5 mM) or high glucose (33 mM) and ARPE-19 and SV40-MES13 cells treated with mannitol (82.5 mM), high glucose (100 mM), or TBFs (5 μg/ml) for 3-7 days were incubated with PI at 37 °C in 5% CO_2_ (v/v) for 15 minutes, according to the manufacturer’s instructions. A Nikon Eclipse Ti inverted microscope was used to identify the PI-positive cells.

### Flow cytometric analyses of cell death

An Annexin V-FITC/PI apoptosis kit (MultiSciences, AP101) was used to quantify apoptotic cells. In brief, HUVECs were treated with apoptosis (Z-VAD-FMK), pyroptosis (disulfiram), ferroptosis (ferrostatin-1), and necroptosis (necrostatin-1) inhibitors and individually incubated with high glucose (33 mM) or mannitol (27.5 mM), and ARPE-19 and SV40-MES13 cells were treated with mannitol (82.5 mM), high glucose (100 mM), or TBFs (5 μg/ml) for 3-7 days. According to the manufacturer’s manual, the cells were trypsinized, washed twice with ice-cold PBS, and incubated with fluorescent dyes for flow cytometric analysis. The levels of apoptosis were determined using a Beckman CytoFlex. Annexin V/PI double-negative cells were considered viable cells, annexin V-positive/PI-negative cells were considered to be early apoptotic cells, and PI-positive cells were considered to be late apoptotic or dead cells.

### Quantitative RT‒PCR analysis

ARPE-19 cells, SV40-MES13 cells, and HUVECs were treated with high glucose and TBF, and total RNA was extracted using an RNA easy extraction kit (Axygen). cDNA was generated using a one-step iScript cDNA synthesis kit (Biomake), and quantitative real-time PCR was performed using EvaGreen qPCR Master Mixes (Abcam) and a CFX96 real-time PCR system (Bio-Rad). Relative quantification was expressed as 2^-∆Ct^, where Ct is the difference between the median Ct value of the triplicate samples and the Ct value of the endogenous RPL19 mRNA control. All primers used for qRT‒PCR analysis are listed in Supplementary Table 3.

### Coimmunoprecipitation and immunoblotting

HEK293T cells were transfected with specified plasmids encoding Myc-, FLAG-, His- or HA-tagged FOXD1, Ub, K48-Ub, K63-Ub, and TRIM21 and lysed in RIPA lysis buffer (Beyotime, P0013C) containing protease inhibitor (Selleck). Cell lysates were then subjected to immunoprecipitation using anti-Flag magnetic beads (Bimake) for transfected proteins. After 3-4 washes with PBST (136.89 mM NaCl, 2.67 mM KCL, 8.1 mM Na_2_HPO_4_, 1.76 mM KH_2_PO_4_, 0.5% Tween 20; pH 7.5), adsorbed proteins were resolved by SDS‒PAGE (Bio-Rad) and subjected to immunoblotting with the indicated antibodies. Cell lysates were also analysed using SDS‒PAGE and immunoblotting to control for protein abundance.

### siRNA-mediated RNA interference

Double-stranded siRNA (RiboBio) targeting human (sequence-1: GAGATCTGTGAGTTCATCAGC, sequence-2: GCGAGATCT GTGAGTTCATCA) or mouse (sequence-1: GCGAGATCTGCGAGTTCAT, sequence-2: CCGAGCTCCGTTTCTAGAT) FOXD1 mRNA were used to silence endogenous FOXD1 expression in ARPE-19 and SV40-MES13 cells. A control siRNA (RiboBio) was used to control for possible nonspecific effects of RNA interference. Cells were transfected with the siRNAs using Lipofect2000 (Invitrogen) reagent for 72 hours before subsequent experiments, and reverse transfection was used to achieve the optimal transfection efficiency.

### Chromatin immunoprecipitation (ChIP)

HEK293T cells transfected with Flag-tagged FOXD1 were harvested and processed with the Pierce Agarose ChIP Kit (26156, Thermo) following the manufacturer’s instructions. In brief, cells were lysed, digested with micrococcal nuclease, and immunoprecipitated with rabbit anti-FLAG® M2 (14793 S, Cell Signaling Technology). Then, protein/DNA complexes were eluted, and cross-linking was reversed after a series of washes. DNA samples were purified using spin columns for quantitative RT‒PCR analysis. All primers used in the ChIP assay are listed in Supplementary Table 3.

### Luciferase reporter assay

HEK293T cells were transfected with the indicated reporters (400 ng) bearing an ORF encoding firefly luciferase, along with pRL-Luc containing the Renilla luciferase ORF as the internal control for transfection and other expression vectors specified in the results section. In brief, cells were lysed in a passive lysis buffer (Promega) 24 hours after transfection. Luciferase assays were performed using a dual luciferase assay kit (Promega), luciferase activity was quantified with a POLARstar Omega microplate reader (BMG Labtech), and firefly luciferase activity was normalized to Renilla luciferase activity as the internal control.

### Immunofluorescence staining and microscopy

To visualize the colocalization of FOXD1 and TRIM21, HEK293T cells were transfected with FOXD1-Flag and TRIM21-Myc, fixed in 4% paraformaldehyde, blocked in 2% bovine serum albumin in PBS for 1 hour, and incubated sequentially with primary antibodies, including anti-Flag (CST, 14793 S, 1:500 dilution) and anti-Myc (CST, 2276 S, 1:500 dilution) antibodies, and Alexa Fluor-488- or Alexa Fluor-594-conjugated secondary antibodies (Jackson, 1:500 dilution). Coverslips were mounted with Vectashield (Vector Labs H-1000), and nuclei were stained with DAPI (Santa Cruz Biotech). Immunofluorescence images were obtained and analysed using a Zeiss LSM710 confocal microscope.

### T2DM mouse model

Mice were bred and maintained in a pathogen-free animal facility at the laboratory animal centre of Wenzhou University. Care of experimental animals was approved by the Wenzhou University Animal Care and Use Committee (approval number WZU-202-010) and was conducted in accordance with Wenzhou University guidelines.

Wild-type C57BL/6 J mice were purchased from Shanghai SLAC Laboratory Animal Co., Ltd. The HFD/STZ-induced T2DM mouse model was established as previously described [81]. Twenty-five mice at 5 weeks of age were fed a HFD for eight weeks. After eight weeks of HFD feeding, mice were fasted overnight and then received intraperitoneal injections of STZ (40 mg·kg^−1^, dissolved in 0.1 mol/L cold citrate buffer, pH 4.2-4.5) daily for five consecutive days. The fasting blood glucose level (after a 6-hour fast) was measured using an automatic analyser kit (Accu-Chek @Active, Roche) and blood samples obtained from the tail vein. Fasting blood glucose levels were measured on the third, fifth, and seventh days following the completion of the five days of STZ injections. The standard for the diabetic mouse model was defined as a fasting blood glucose level maintained above 16.7 mmol/L. As a control group, nondiabetic (ND) mice were fed a normal fat diet and intraperitoneally administered sodium citrate buffer instead of STZ. Only successfully established diabetic mice were included in the subsequent experiments; those that failed to develop the desired model were excluded. Prior to commencing subsequent animal studies, mice were randomly allocated to distinct experimental groups. For treatment, TBFs (300 mg/kg dissolved in 1% (v/v) carboxymethyl cellulose sodium in PBS) or carboxymethyl cellulose sodium was administered intragastrically to HFD/STZ-induced T2DM or ND mice every day for four weeks as follows: ND group (ND mice, daily administration of 1% (v/v) carboxymethyl cellulose sodium in PBS), TBF group (ND mice, daily administration of 300 mg/kg TBFs), T2DM group (T2DM mice, daily administration of 1% (v/v) carboxymethyl cellulose sodium in PBS), and T2DM-TBF group (T2DM mice, daily administration of 300 mg/kg TBFs). The body weight and fasting blood glucose concentrations were recorded weekly for four weeks. The investigators were not blinded during the experiments. At the end of four weeks following the onset of HFD/STZ-induced diabetes, all mice were euthanized to collect serum, eyeballs, and kidneys for subsequent analysis.

### Analysis of serum biochemical indices

Total cholesterol, high-density lipoprotein cholesterol, and triglycerides were measured with assay kits (Nanjing Jiancheng Bioengineering Institute), and ELISA kits (Lianke Biotech) were used to measure insulin, IL-6, TNF-α, and IL-1β concentrations in serum.

### Histology and immunohistochemistry

For histological analyses, eye and kidney samples were harvested from mice and fixed in 4% paraformaldehyde for 24 hours at 4 °C, dehydrated in a graded ethanol series, embedded in paraffin, and sliced into 6-μm sections, which were then stained with haematoxylin and eosin (H&E). For IHC analysis, retinal and renal samples were harvested and fixed in 4% paraformaldehyde for 24 hours at 4 °C, dehydrated in a graded ethanol series, and embedded in paraffin. Samples sectioned at a thickness of 6 μm were washed twice with PBS and permeabilized with 0.5% Triton X-100 for 5 minutes, blocked in 3% bovine serum albumin in PBS for 30 minutes, and incubated sequentially with primary antibodies, including anti-FOXD1 (Thermo, PA5-35145, 1:100 dilution), anti-BCL-2 (Abcam, ab182858, 1:100 dilution), and anti-cleaved caspase 3 (CST, 9664 S, 1:100 dilution) antibodies. After incubation with the corresponding secondary antibodies (ZSGB-Bio, Beijing, 1:1000 dilution) at room temperature for 1 hour, the sections were treated with a peroxidase DAB substrate kit (Vector Laboratories, Inc. Burlingame, CA) and counterstained with haematoxylin. All quantitative analyses were performed using ImageJ software.

### TUNEL assay

Eye and kidney samples were harvested from mice and fixed with 4% PFA in PBS. The TUNEL assay was performed using a Cell Death Detection Kit (Ruchuang, RCT-50R). TUNEL images were obtained and analysed using a Nikon Eclipse Ti inverted microscope or a Zeiss LSM880 confocal microscope.

### RNA-seq analysis

The quantity and integrity of RNA were assessed using a K5500 (Beijing Kaiao, China) and an Agilent 2200 TapeStation (Agilent Technologies, USA) separately. Paired-end reads were aligned to the human reference genome hg38 with HISAT2. HTSeq v0.12.4 was used to count the reads mapped to each gene. The abundance of each RNA was quantified as the fragments per kilobase of exon per million mapped fragments (FKPM) value using Cufflinks v2.2.1. Genes with FPKM < 1 in all samples were excluded, and for the remaining genes, FPKM values of less than 1 were set to 1 in downstream analyses. The polysome-seq data were deposited in the NCBI Gene Expression Omnibus database under accession code 241638.

### Mass spectrometry analysis

Nanoscale liquid chromatography coupled to tandem mass spectrometry for protein identification, characterization, and label-free quantification was performed by OE Biotechnology Company (Shanghai). Tryptic peptides were separated on a C18 column and analysed with an LTQ-Orbitrap Velos system (Thermo). Proteins were identified using the search engine of the NCBI against the human or mouse RefSeq protein databases. The mass spectrometry data of the FOXD1-interacting proteins are provided in Supplementary Table 4.

### Statistical analysis and reproducibility

Quantitative data are presented as the mean ± standard error of the mean (SEM) of at least three independent experiments. When appropriate, the statistical significance of differences among more than two comparison groups was analysed using one-way ANOVA with the Bonferroni correction for multiple comparisons. Differences were considered significant at *p* < 0.05. All samples were included in the analyses if preserved and properly processed, and the preestablished standard excluded no samples or animals. No statistical method was used to predetermine the sample size, and no experiments except those involving animals were randomized. All experiments were repeated a minimum of three times independently with similar results to ensure reproducibility. The investigators were not blinded to the group allocations during the experiments and outcome assessments.

### Reporting summary

Further information on research design is available in the Nature Research Reporting Summary linked to this article.

### Supplementary information

Supplemental Figures__CDDIS-22-5026RR

Supplementary Table 1_Plasmid Information

Supplementary Table 2_Antibody Information

Supplementary Table 3_Oligo Information

Supplementary Table 4_The Mass Spectrometry Data

Original Data File

Reporting Summary

## Data Availability

All experimental data sets generated and analysed during the current study are included in this published article and its supplementary information files. Additional data and further information are available from the corresponding author upon reasonable request.
