# Supplementary material for: The TRIM21-FOXD1-BCL-2 axis underlies hyperglycaemic cell death and diabetic tissue damage
Source: Cell Death Dis. 2023 Dec 13;14(12):825. doi: 10.1038/s41419-023-06355-1 (PMC10719266; doi:10.1038/s41419-023-06355-1)
Supplement: Supplementary file 1 — Supplemental Figures__CDDIS-22-5026RR [file 41419_2023_6355_MOESM1_ESM.pdf]

Supplementary Figure 1

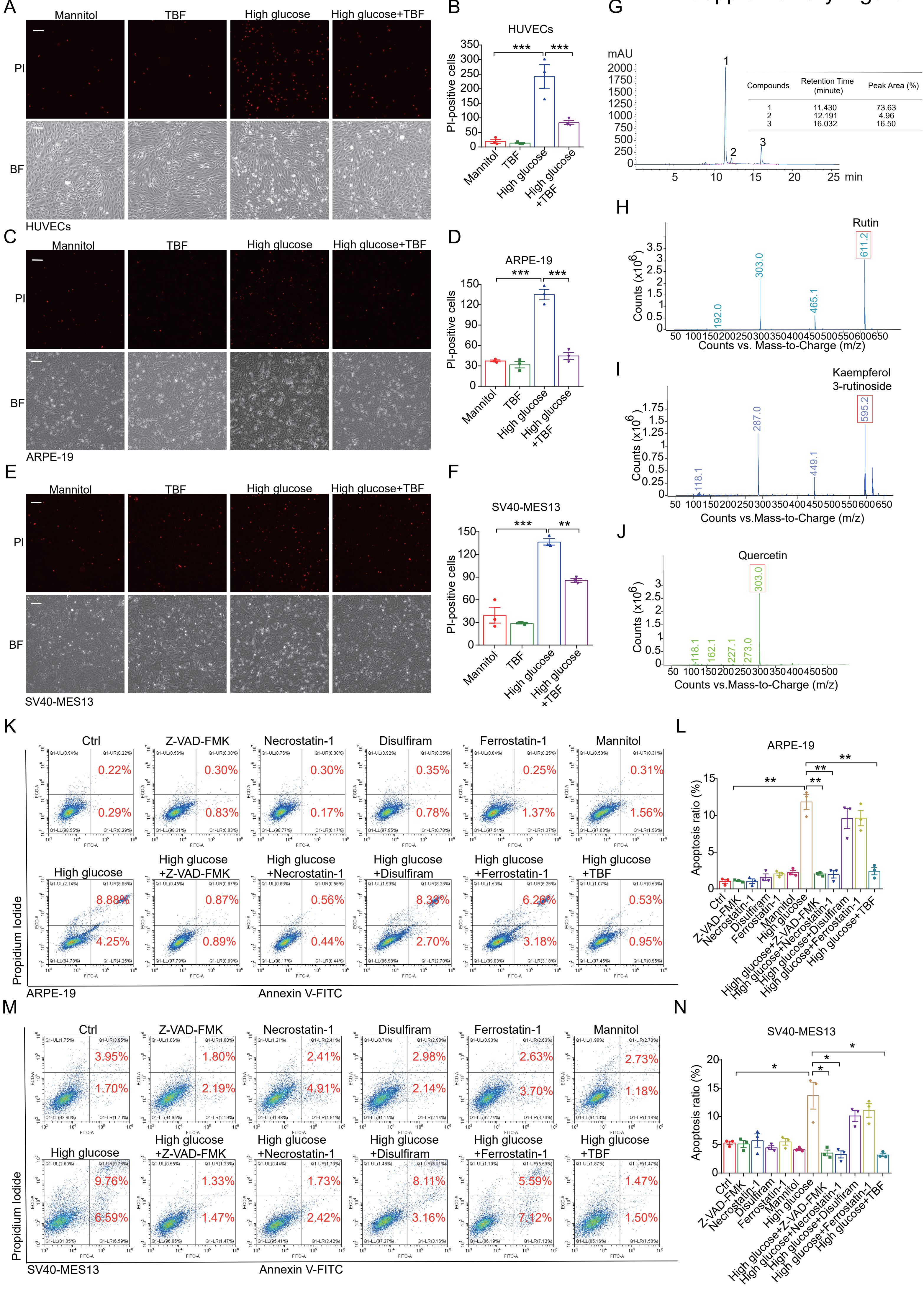

Supplementary Figure 1, corresponding to Figure 1. **(A-B),** Primary HUVECs cultured in high glucose medium (33 mM) for 72 hours showed a significant cell death, revealed by propidium iodide (PI) staining via fluorescence microscopy (A). Quantitative analysis of PI-positive cells (B). Treatment with TBF at 5 μg/ml exhibited substantial protection against hyperglycaemia-induced HUVEC apoptosis. n=3. **(C-D),** Hyperglyacemia-induced cell apoptosis was also apparent in ARPE-19, revealed by PI staining via fluorescence microscopy, and TBF protected against cell death. n=3. **(E-F),** Hyperglycaemia-induced cell apoptosis was also apparent in SV40-MES13, revealed by PI staining via fluorescence microscopy, and TBF protected against cell death. n=3. **(G-J),** The separation and identification of Tartary buckwheat flavonoids (TBF) extract. **(G)**, HPLC chromatogram revealed the major components of TBF. Mass spectrometry (MS) analyses showed the ion fragmentation chromatogram of compounds 1–3 which were identified as rutin **(H)**, kaempferol 3-rutinoside **(I)**, and quercetin **(J)**, respectively. **(K-L),** Inhibitors of apoptosis (Z-VAD-FMK), necroptosis (necostatin-1), pyroptosis (disulfiram), and ferroptosis (ferrostatin-1) were individually incubated together with high glucose (100 mM) in ARPE-19 for 120 hours. n=3. **(M-N),** Inhibitors of apoptosis (Z-VAD-FMK), necroptosis (necostatin-1), pyroptosis (disulfiram), and ferroptosis (ferrostatin-1) were individually incubated together with high glucose (100 mM) in SV40-MES13 for 120 hours. n=3. Unless otherwise specified, n=3 independent experiments (mean ± SEM); *, $P<0.05$, **, $P<0.01$, ***, $P<0.001$, and ****, $P<0.0001$ by statistical analysis of the indicated comparison with ANOVA and Bonferroni correction.

Supplementary Figure 2

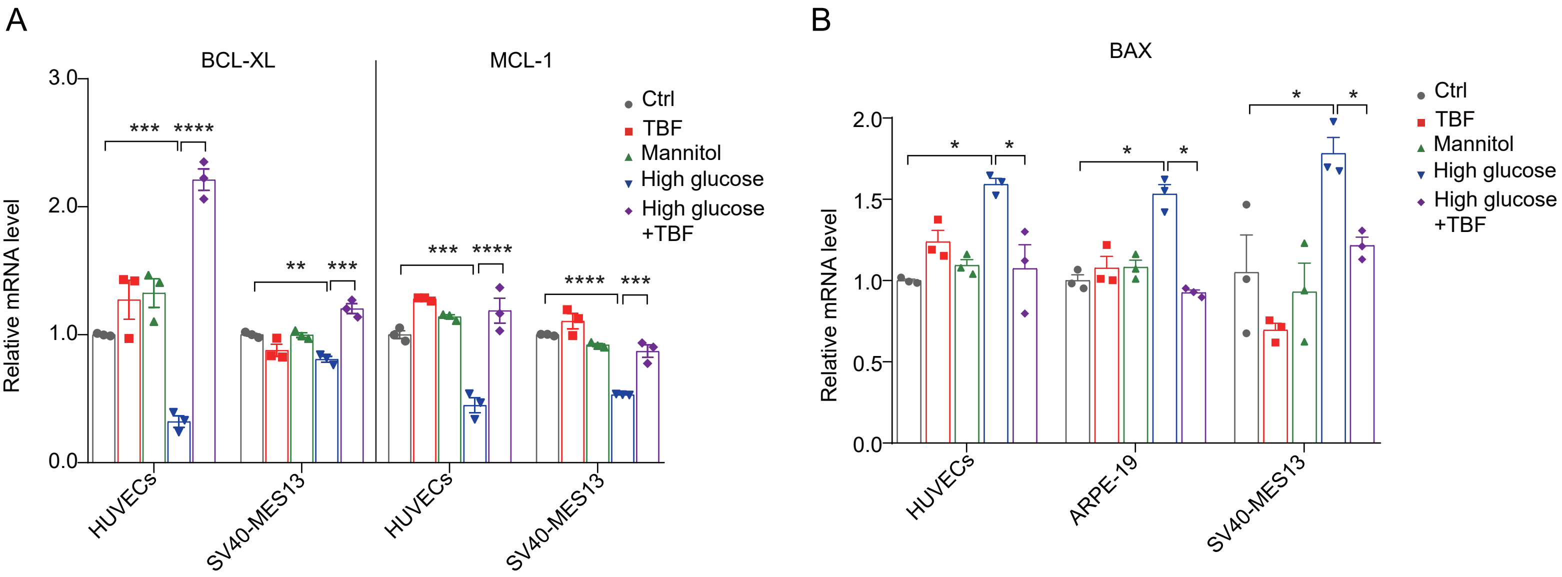

Supplementary Figure 2, corresponding to Figure 2. **(A),** RT-qPCR assay revealed that high glucose treatment downregulated the mRNA expressions of BCL-XL and MCL-1 in venous and renal cells, which were reversed by TBF. n=3. **(B),** RT-qPCR assay revealed that high glucose treatment upregulated the mRNA expression of BAX in venous, retinal, and renal cells, while this upregulation was reversed by TBF. n=3.

A

Proteins	PSMs
ACTC1	18
RPS27A	4
TRIM21	3
PRKDC	37
TAOK3	1
STK38	1
CSNK2A2	1
PTPN23	1
HSP90AB1	1
EWSR1	4
RBM14	2

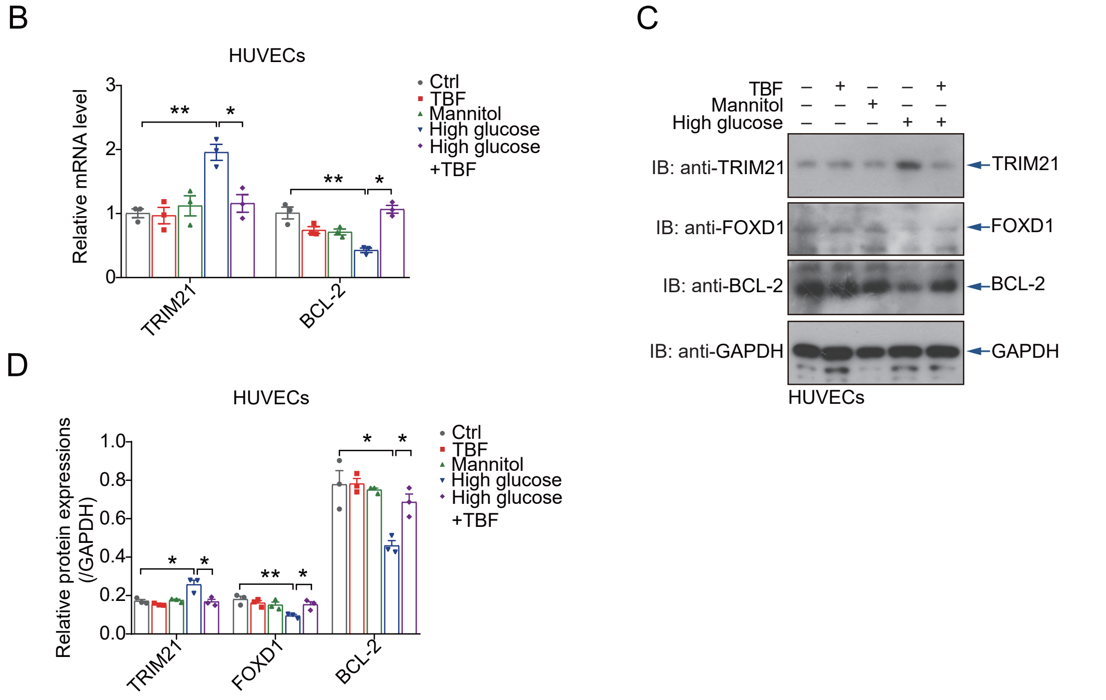

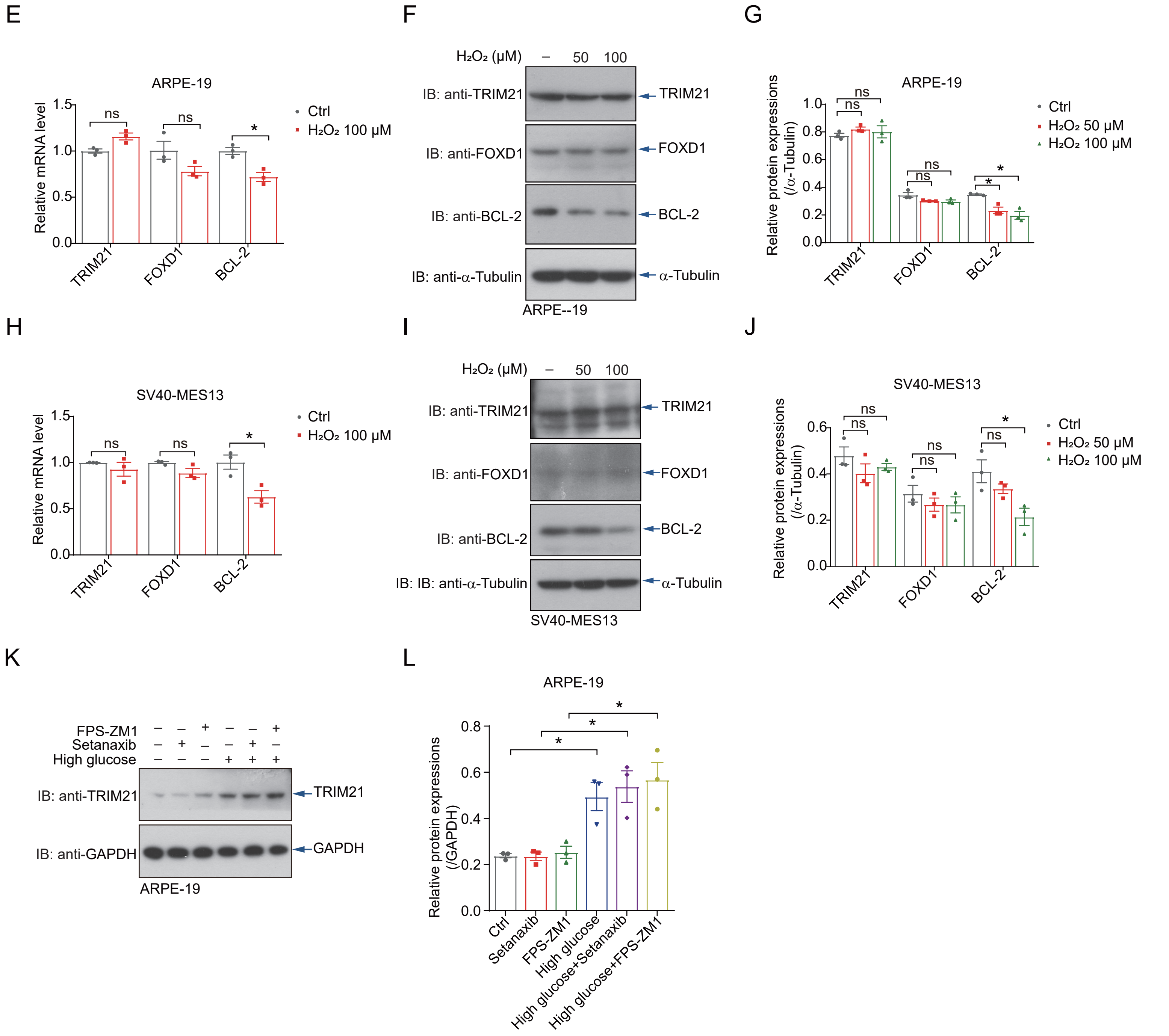

Supplementary Figure 3, corresponding to Figure 4. (A), A proportion of interacting proteins of FOXD1 were identified by mass spectrometry. **(B-D),** High glucose culture upregulated both mRNA (B) and protein (C) levels of TRIM21 in HUVCs cells, a process was reversed by TBF treatment. Quantitative analysis of the relative protein expression were shown (D). n=3. **(E-G),** Treatment with H_2O_2 at 50 μM and 100 μM for 48 hours did not exert regulatory effects on the mRNA (E) and protein (F) levels of TRIM21 and FOXD1 in ARPE-19. However, it decreased both the mRNA and protein levels of BCL-2. Quantitative analysis of the relative protein expression were shown (G). n=3. **(H-J),** Treatment with H_2O_2 at 50 μM and 100 μM for 48 hours did not exert regulatory effects on the mRNA (H) and protein (I) levels of TRIM21 and FOXD1 in SV40-MES13. Quantitative analysis of the relative protein expression were shown (J). n=3. **(K-L),** ARPE-19 cells cultured in high glucose medium (200 mM) for 72 hours showed a significant increase in TRIM21 protein levels, which were not reversed by treatment with RAGE inhibitor (FPS-ZM1, 10 μM, 72 hours) or ROS inhibitor (Setanaxib, 10 μM, 72 hours), as revealed by immunoblotting analysis (K). Quantitative analysis of the expression of TRIM21 protein was shown (L). n=3.

Supplementary Figure 4

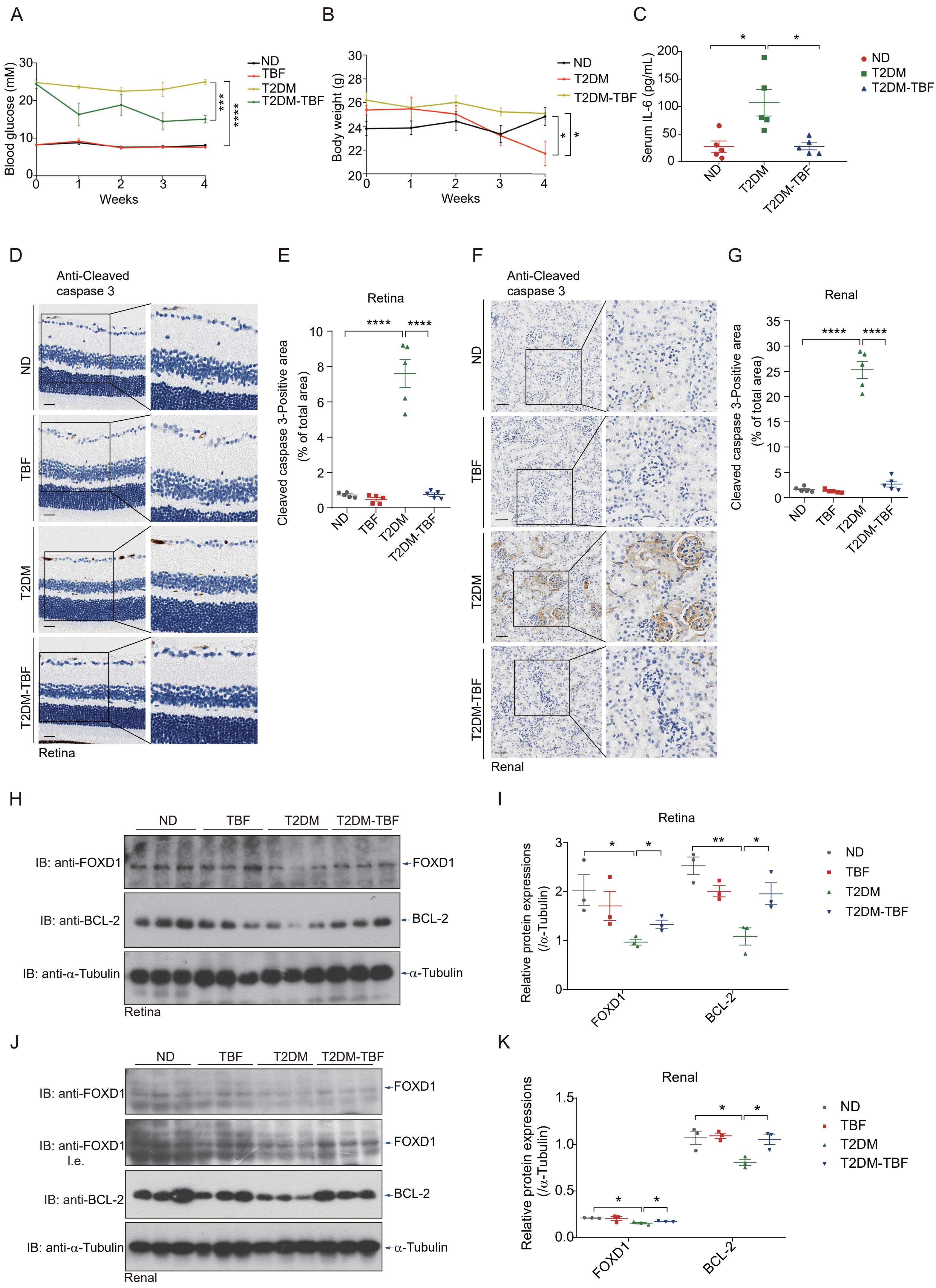

Supplementary Figure 4, corresponding to Figure 5. **(A),** Blood glucose levels were measured in T2DM mice once a week, with or without TBF treatment. n=5. **(B),** Body weights were measured in T2DM mice once a week, with or without TBF treatment. n=5. **(C),** Serum IL-6 was measured by ELISA analysis in T2DM mice, with or without TBF treatment. n=5. **(D-G),** IHC staining indicated that cleaved caspase 3 protein level was increased in the retina (D-E) and renal (F-G) with hyperglycaemic injuries. TBF treatment reversed this process. n=5. Scale bars=50 μm (left panels of D) or Scale bars=100 μm (left panels of F). **(H-K),** Immunoblotting analysis indicated that protein levels of FOXD1 and BCL-2 were decreased in the retinal (H-I) and renal (J-K) with hyperglycaemic injuries, TBF treatment reversed this process. n=3.
