# Supplementary material for: The TRIM21-FOXD1-BCL-2 axis underlies hyperglycaemic cell death and diabetic tissue damage
Source: Cell Death Dis. 2023 Dec 13;14(12):825. doi: 10.1038/s41419-023-06355-1 (PMC10719266; doi:10.1038/s41419-023-06355-1)

Figure 1I

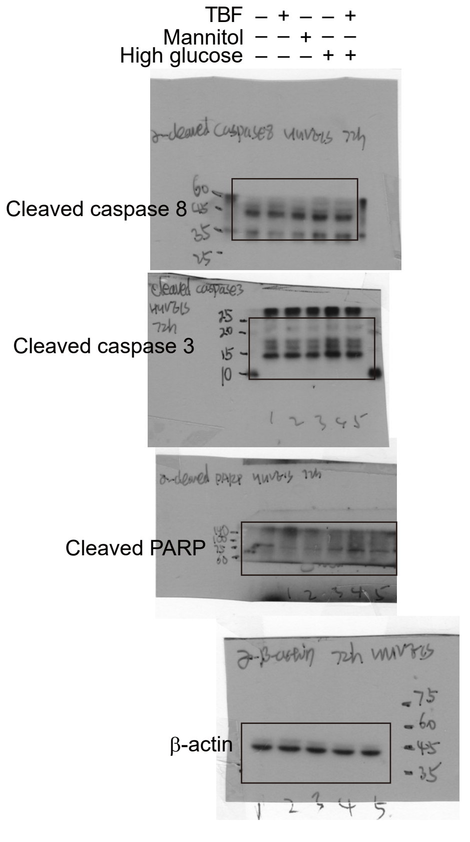

Figure 1K

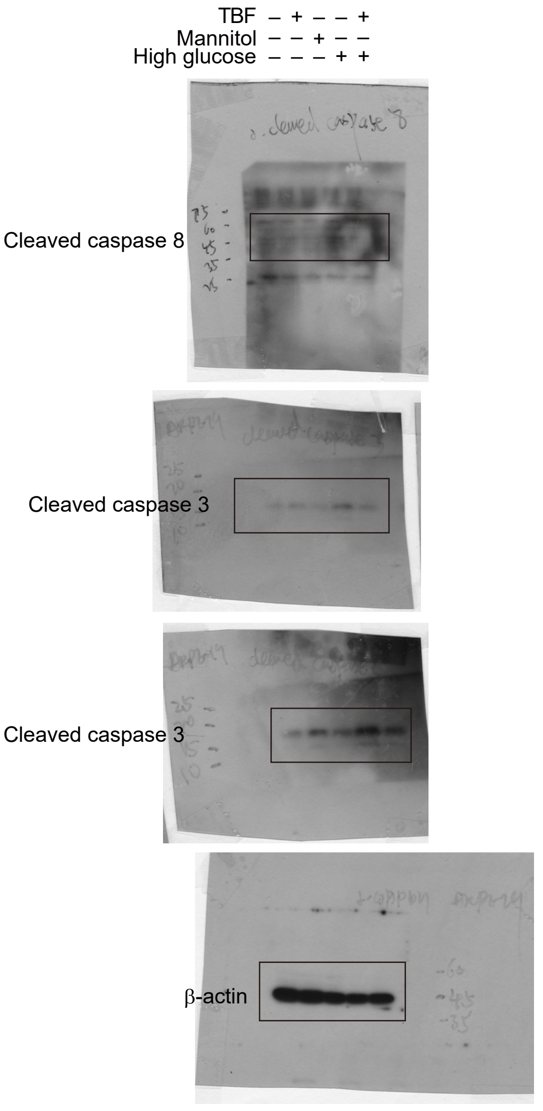

Figure 1M

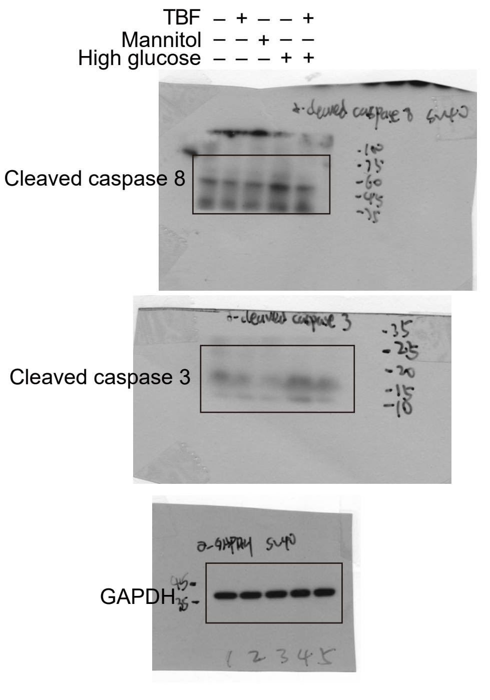

Figure 2E

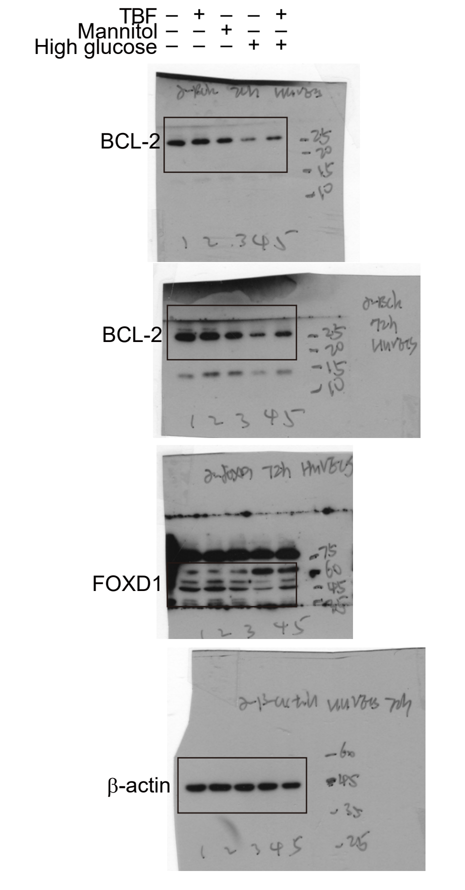

Figure 2G

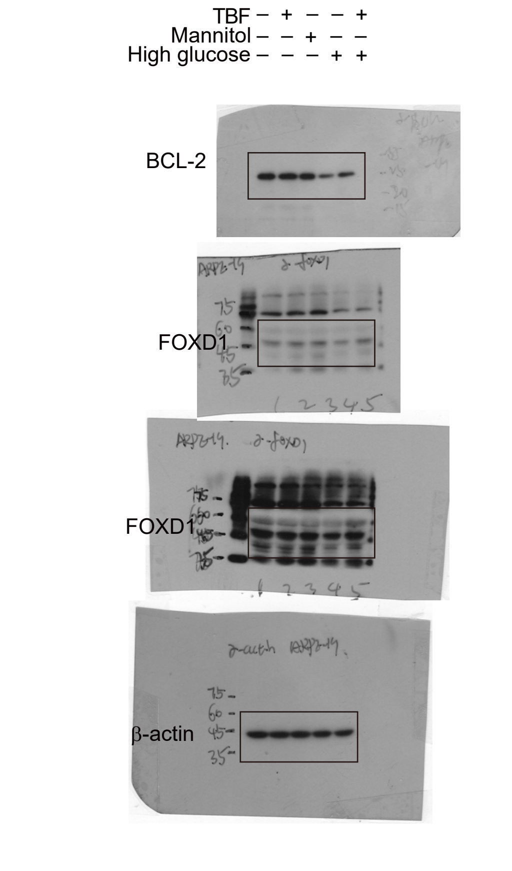

Figure 2I

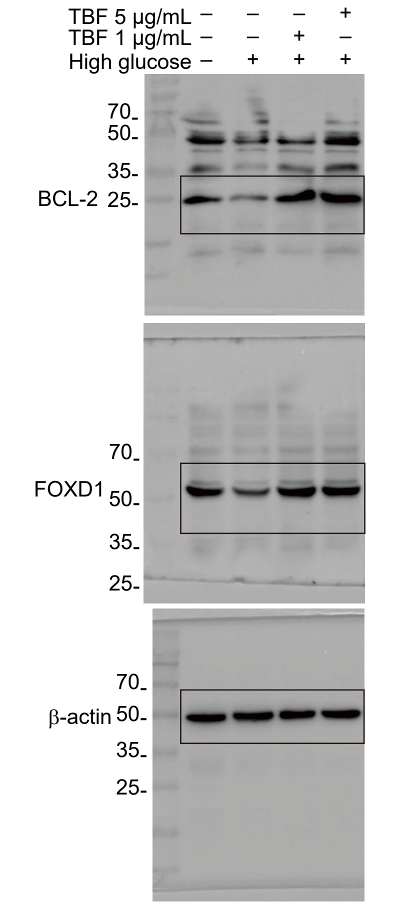

Figure 3C

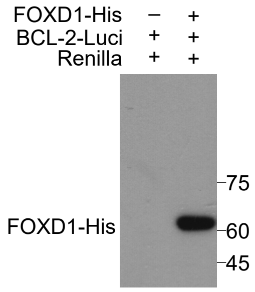

Figure 3D

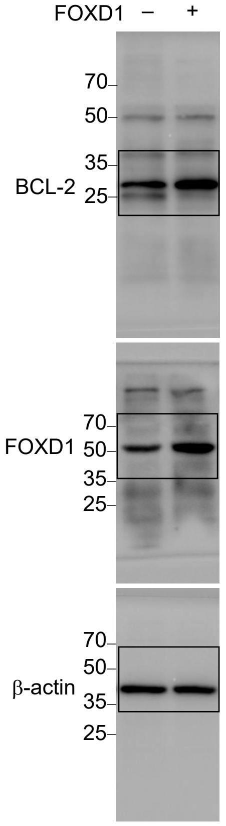

Figure 3F

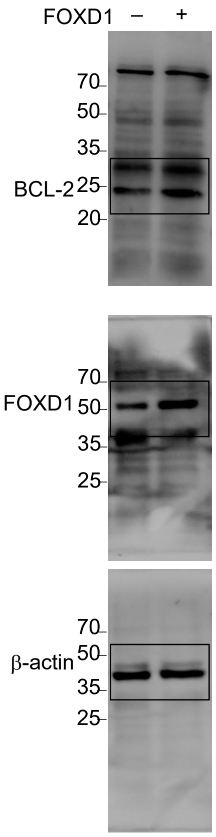

Figure 3H

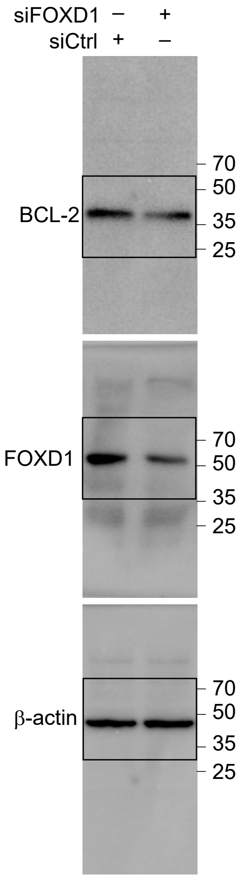

Figure 3J

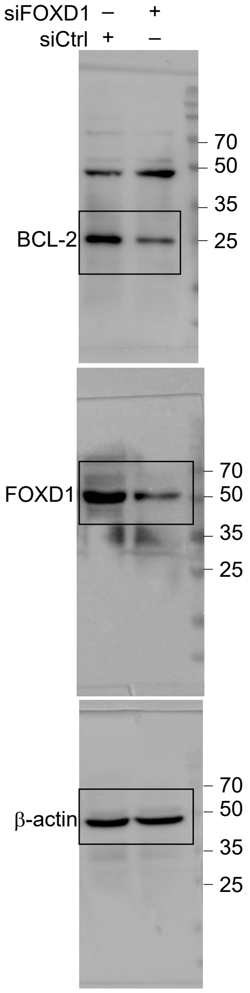

Figure 3L

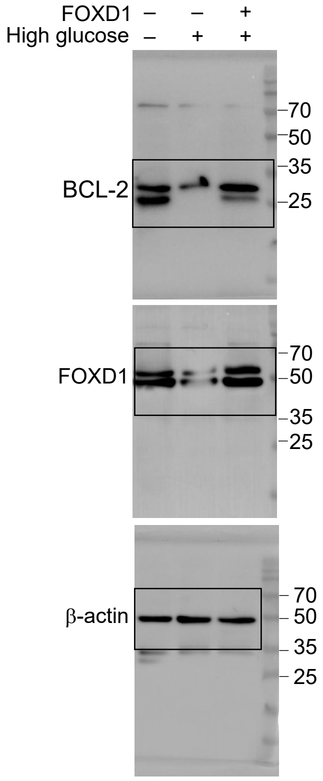

Figure 3N

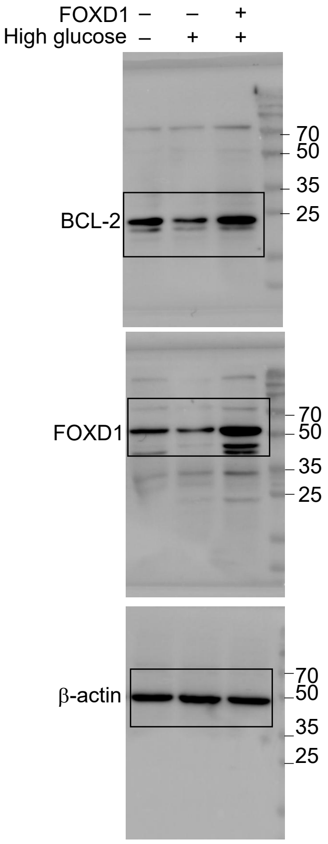

Figure 4A

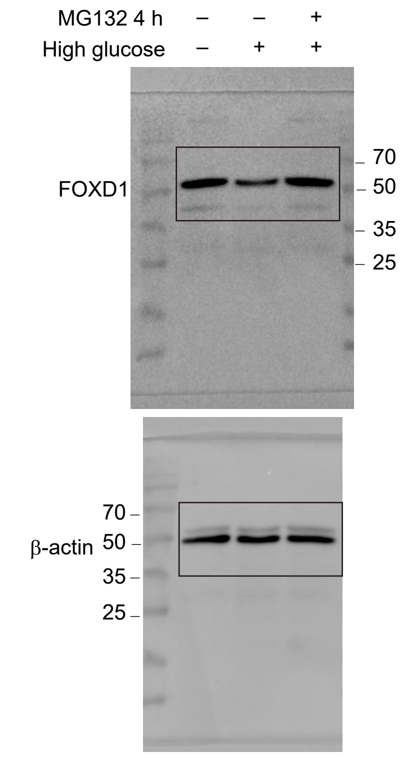

Figure 4C

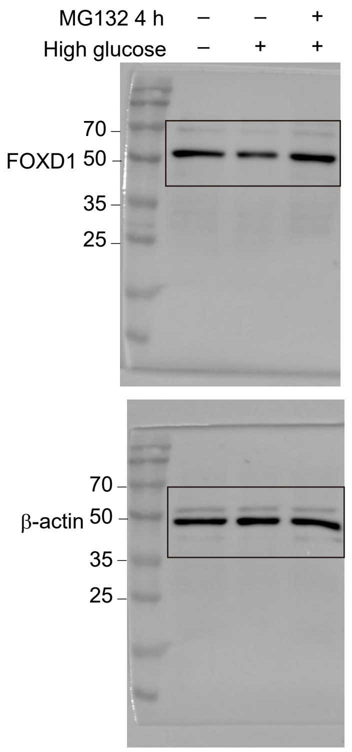

Figure 4F

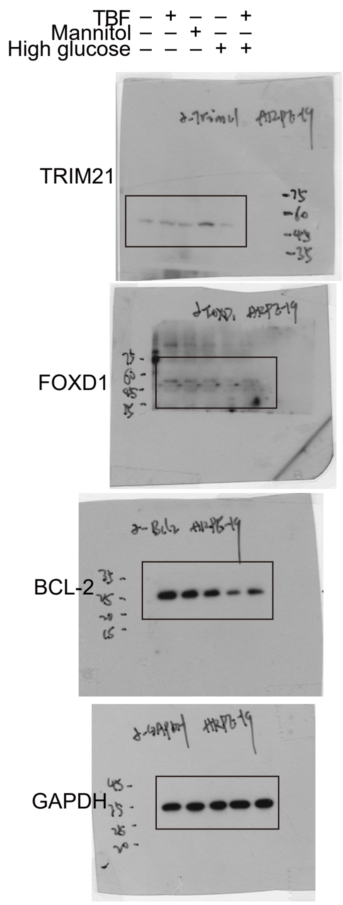

FIgure 4I

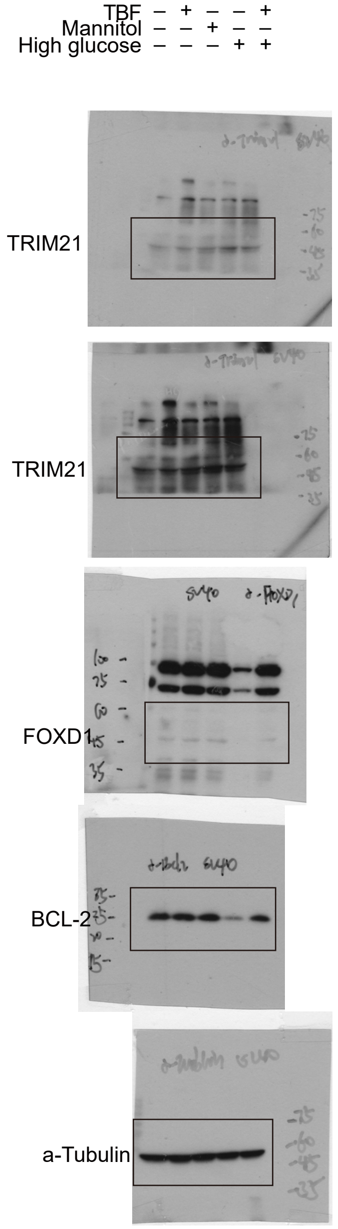

Figure 4K

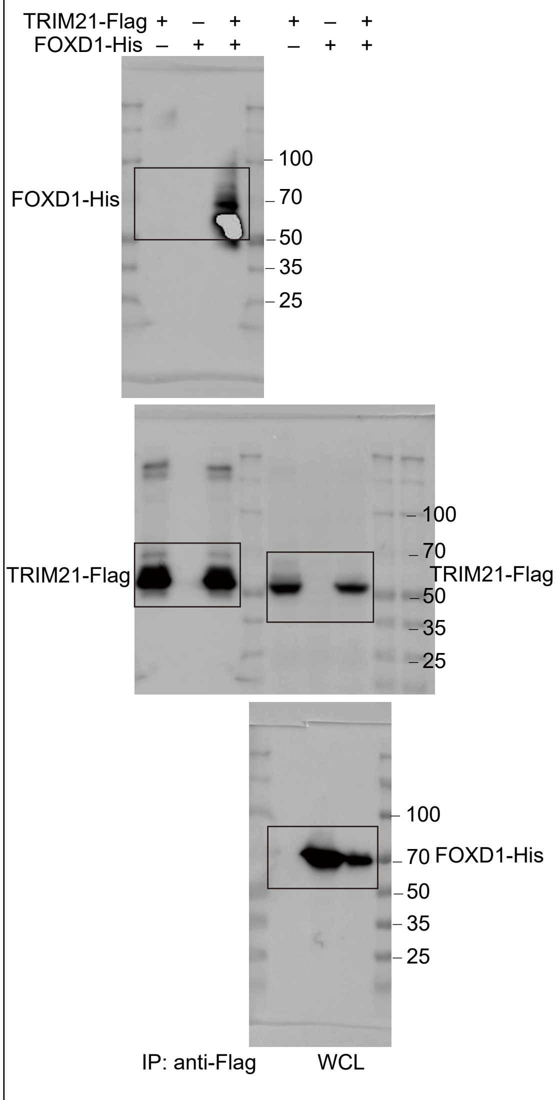

Figure 4L

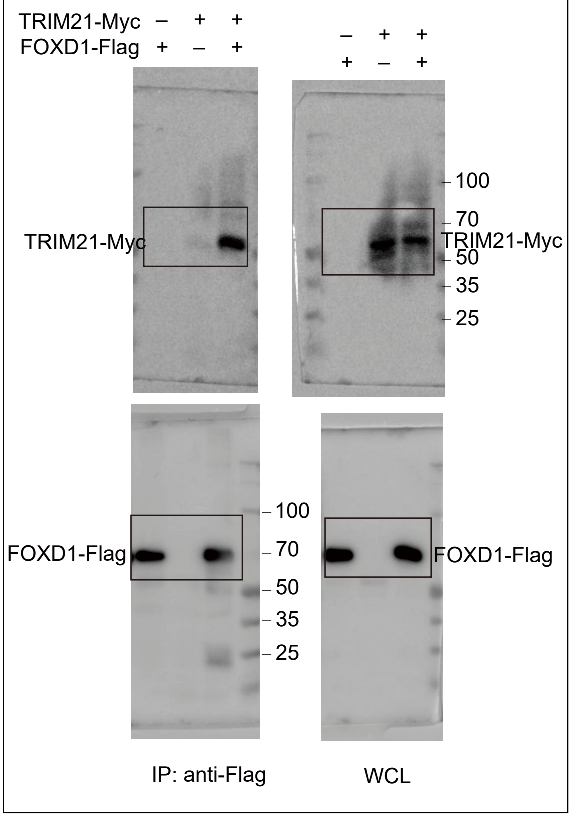

Figure 4N

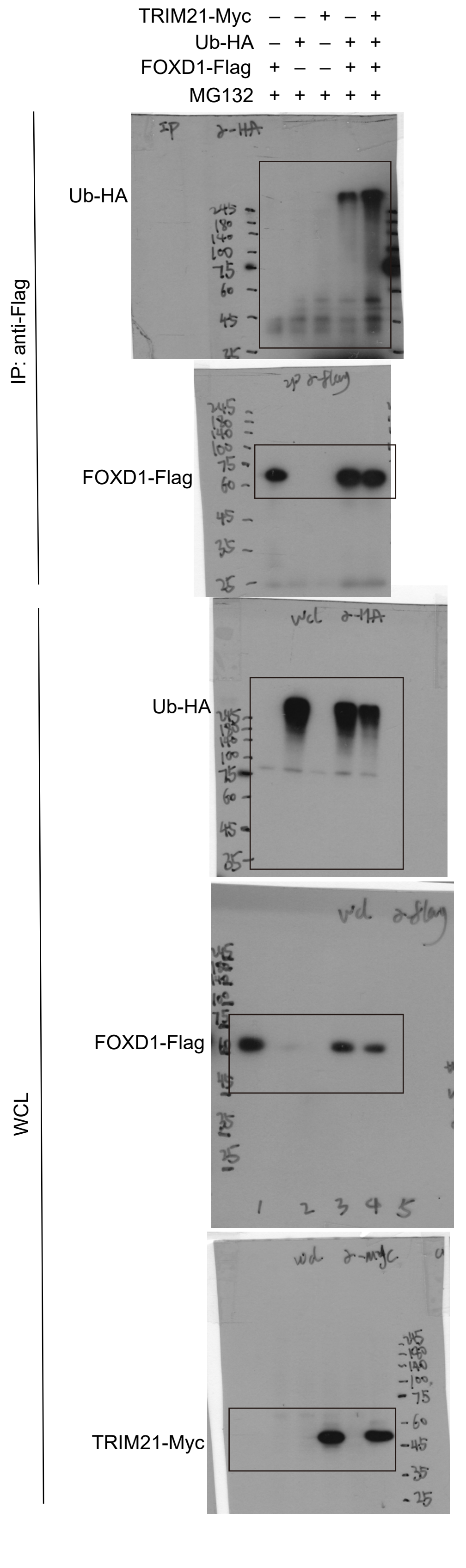

Figure 4O

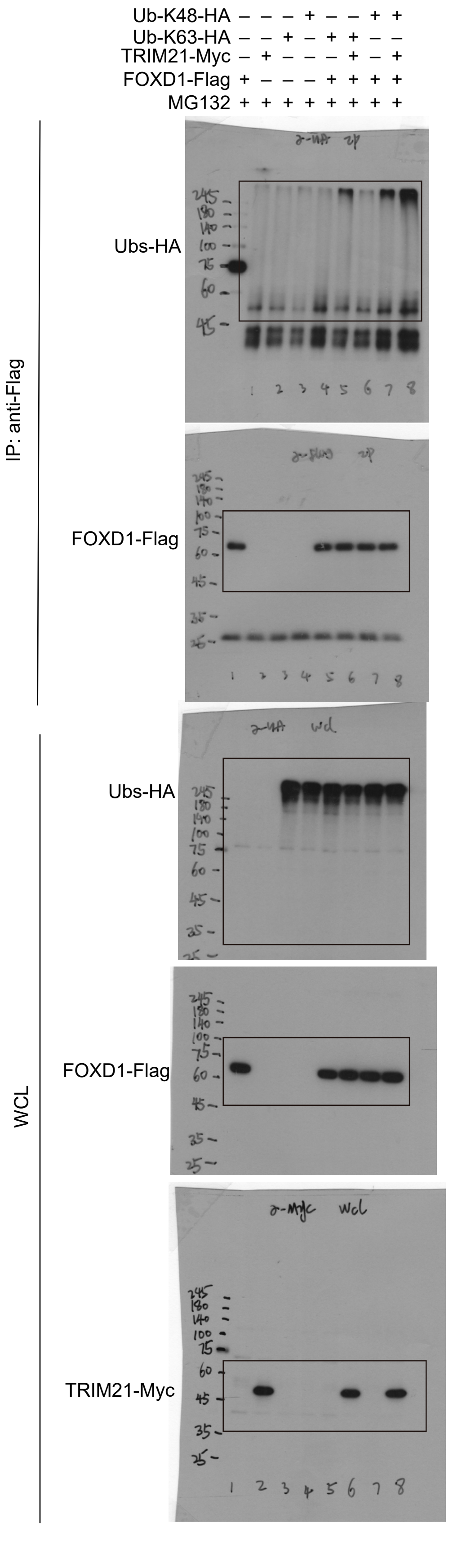

Figure 4P

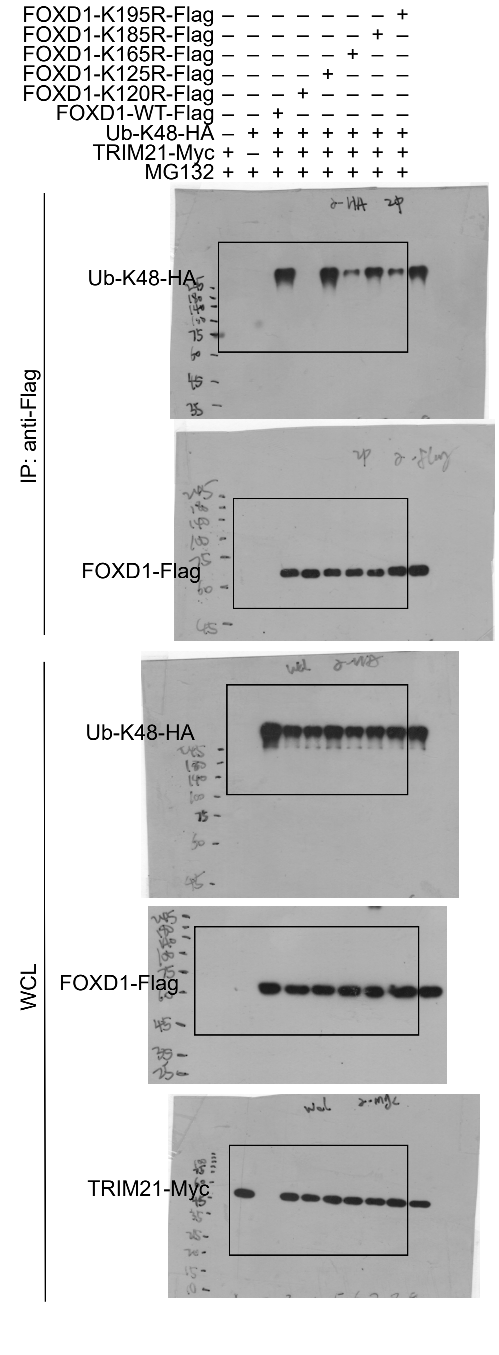

Supplementary Figure 3C

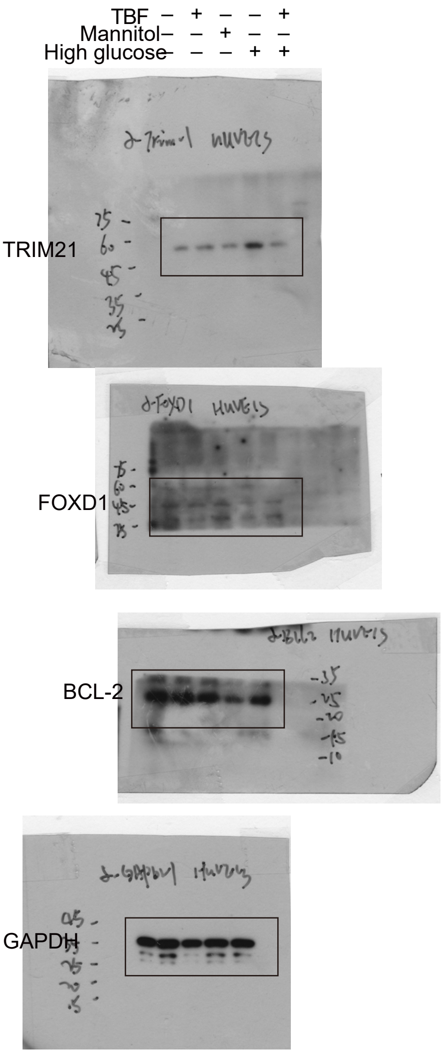

Supplementary Figure 3F

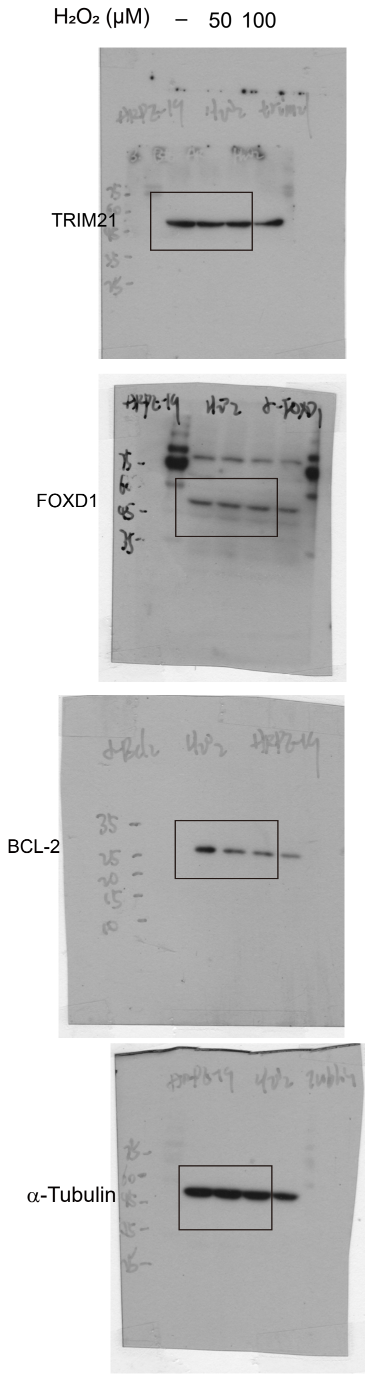

Supplementary Figure 3I

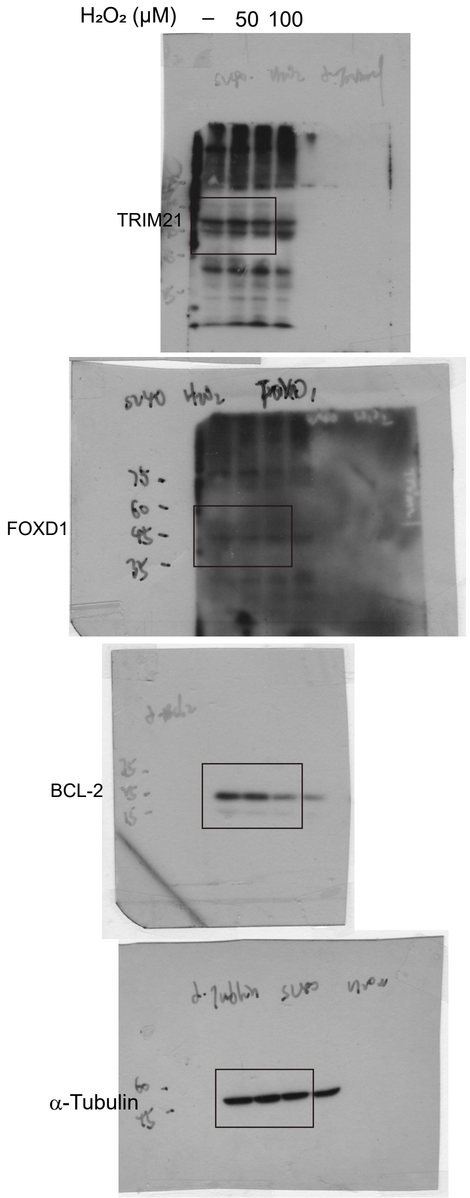

Supplementary Figure 3K

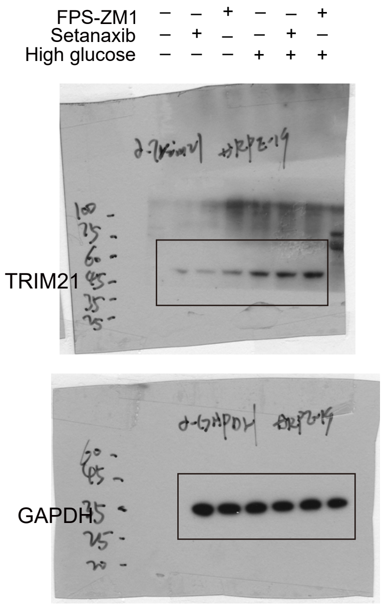

Supplementary Figure 4H

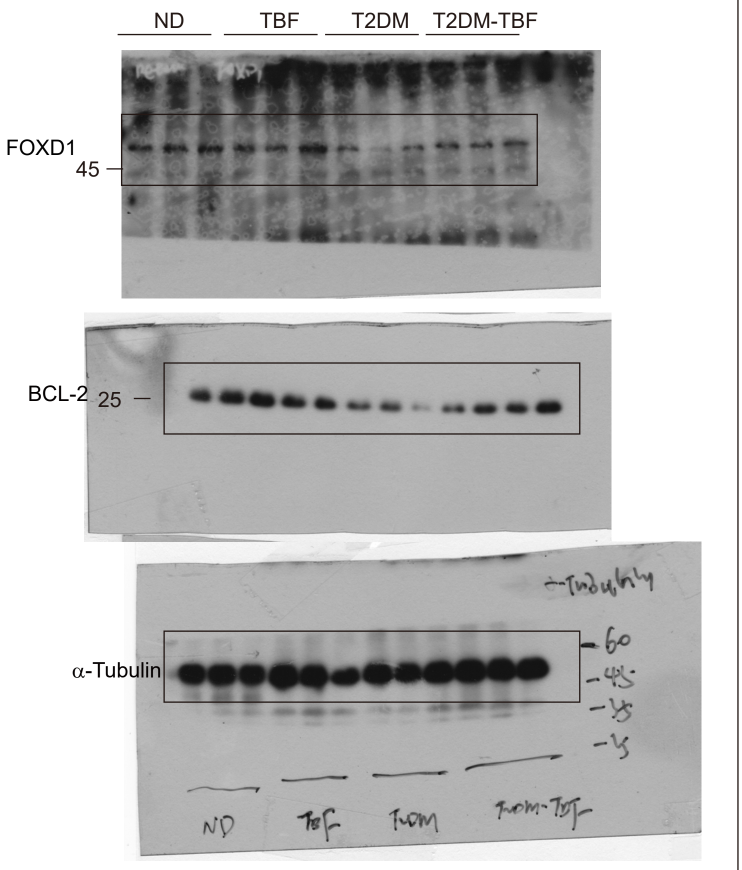

Supplementary Figure 4J

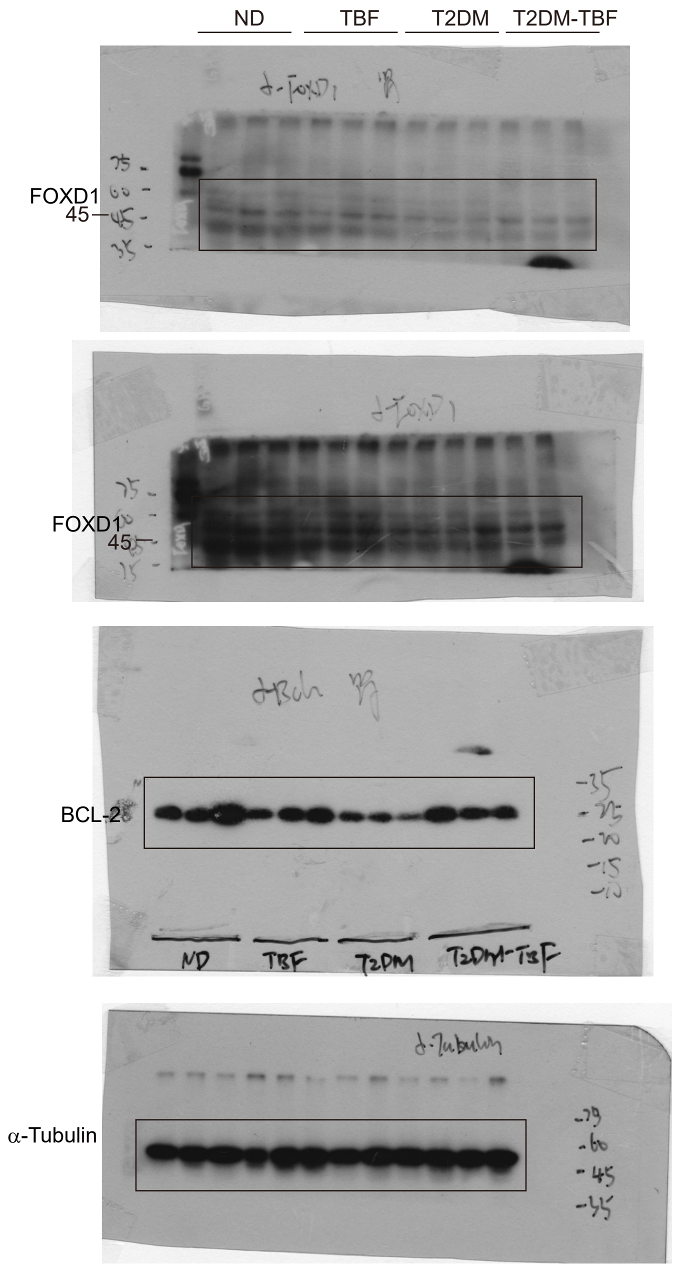

Supplement: Supplementary file 6 — Original Data File [file 41419_2023_6355_MOESM6_ESM.pdf]
